# Integrating Life-Cycle Assessment (LCA) and Artificial Neural Networks (ANNs) for Optimizing the Inclusion of Supplementary Cementitious Materials (SCMs) in Eco-Friendly Cementitious Composites: A Literature Review

**DOI:** 10.3390/ma18184307

**Published:** 2025-09-14

**Authors:** A. Arvizu-Montes, Oswaldo Guerrero-Bustamante, Rodrigo Polo-Mendoza, M.J. Martinez-Echevarria

**Affiliations:** 1 Department of Construction Engineering and Projects of Engineering, Construction Engineering Laboratory of the University of Granada (LabIC.UGR), 18071 Granada, Spain; 2 Department of Civil & Environmental, Universidad de la Costa, Barranquilla 080002, Colombia; 3 Department of Civil & Environmental Engineering, Universidad del Norte, Barranquilla 080003, Colombia; 4 Faculty of Science, Charles University, 128 00 Prague, Czech Republic

**Keywords:** artificial neural networks, cement sustainability, life-cycle assessment, low-carbon designs, machine learning, supplementary cementitious materials

## Abstract

The construction industry is a major contributor to global environmental impacts, particularly through the production and use of cement-based materials. In response to this challenge, this study provides a comprehensive synthesis of recent advances in the integration of Life-Cycle Assessment (LCA) and Artificial Neural Networks (ANNs) for optimizing cementitious composites containing Supplementary Cementitious Materials (SCMs). A total of 14 case studies specifically addressing this topic were identified, reviewed, and analyzed, spanning various binder compositions, ANN architectures, and LCA frameworks. The findings highlight how hybrid ANN–LCA systems can accurately predict mechanical performance while minimizing environmental burdens, supporting the formulation of low-carbon, high-performance cementitious composites. The diverse SCMs explored, including fly ash, slag, silica fume, waste glass powder, and rice husk ash, demonstrate significant potential for reducing CO_2_ emissions, energy consumption, and raw material depletion. Furthermore, the systematic comparative matrix developed in this work offers a valuable reference for researchers and practitioners aiming to implement intelligent, eco-efficient mix designs. Overall, this study contributes to advancing digital sustainability tools and reinforces the viability of ANN–LCA integration as a scalable decision-support framework for green construction practices.

## 1. Introduction

**Figure 1:**
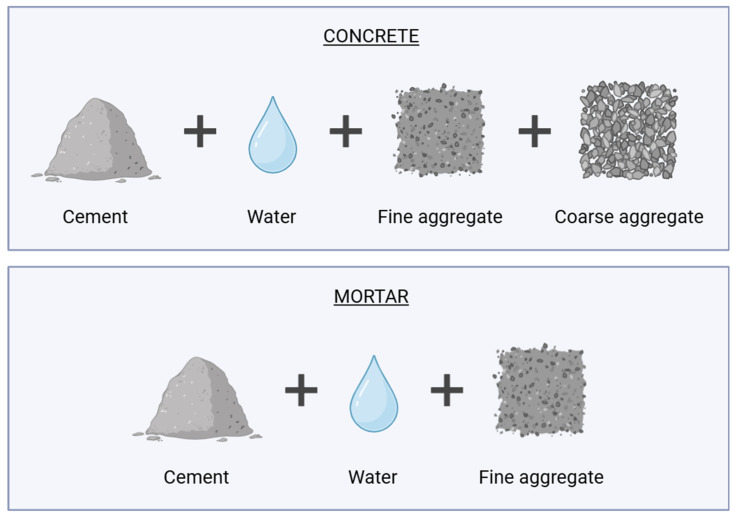
Compositional differences between mortar and concrete.

Cementitious composites are construction materials composed primarily of hydraulic cement (commonly Portland cement) combined with aggregates (i.e., sand and/or gravel), water, and often fillers and other additives/admixtures to enhance performance [1,2]. These composites, such as mortar and concrete (see Figure 1), are indispensable in the construction industry due to their wide availability, adaptability, and excellent mechanical properties, making them foundational to infrastructure development, urbanization, and economic growth worldwide [3,4,5]. However, their environmental burdens are elevated because cement production is highly energy-intensive and responsible for approximately 5–7% of global anthropogenic CO_2_ emissions, mainly due to the calcination of limestone and the combustion of fossil fuels [6,7,8]. In this regard, it is essential to redesign cementitious composites to be more eco-friendly, thus mitigating the impacts of climate change and safeguarding non-renewable resources [9,10]. A key strategy in achieving this sustainability goal is the incorporation of Supplementary Cementitious Materials (SCMs), which include industrial by-products like fly ash, silica fume, and ground granulated blast furnace slag, as well as natural pozzolans and agricultural residues such as rice husk ash [11,12]. SCMs not only partially replace cement, thus lowering air emissions (e.g., CO_2_, NO_x_, SO_2_, CO, dioxins, furans, and trace heavy metals, among others) and conserving virgin raw materials but also often improve the durability and long-term strength of the composites when properly engineered [13,14].

Designing cementitious composites incorporating SCMs is inherently challenging due to the complex interplay between mechanical performance and environmental impact, where varying SCM dosages can either enhance or compromise compressive strength, durability, and sustainability outcomes [15,16]. Excessive SCM content may lower greenhouse gas emissions by reducing clinker usage, but it can also degrade mechanical properties, potentially increasing the total material required and offsetting environmental benefits, whereas insufficient SCM use fails to capitalize on potential sustainability gains [17,18]. Thus, refining raw material dosages is essential to achieve a mixture design that balances mechanical performance with potential environmental savings [19,20,21]. In this regard, the Life-Cycle Assessment (LCA) methodology has become a widely adopted approach for quantifying the environmental burdens of these materials, including (but not limited to) air emissions, energy demand, and water use [4]. Nonetheless, identifying a replacement level that simultaneously minimizes environmental detriments across multiple impact categories while maintaining the required mechanical performance is complex, especially given the variable nature of different SCMs and their physicochemical interactions with other raw materials [7,22]. Whereas some well-established materials, such as slag or fly ash, have regulated limits to ensure performance and safety, defining the ideal proportions remains a challenge for novel, innovative, or less standardized SCMs. To address this, Artificial Neural Networks (ANNs) have gained prominence as powerful tools capable of modeling nonlinear relationships and optimizing mix designs by learning from experimental data, thereby facilitating the rapid identification of optimal SCM proportions that meet both mechanical and environmental performance objectives [23,24].

Integrating LCA and ANNs for refining the inclusion of SCMs in sustainable cementitious composites has become an emerging trend to address the complex challenge of simultaneously improving environmental performance and mechanical properties [25,26]. While various case studies have been conducted to demonstrate the potential of this synergy [24,27,28], a unified framework or comprehensive synthesis is lacking, which hinders the understanding of these isolated research efforts in a cohesive manner. The diversity in methodologies, regional material variability, and inconsistent performance indicators has led to a fragmented body of knowledge without a clear consensus. Therefore, this literature review aims to systematically examine the current state-of-the-art related to LCA–ANN integration in SCM-based cementitious composite mix optimization, uncover gaps and methodological limitations, and provide a critical analysis that highlights both the strengths and unresolved challenges in the field. Additionally, the present study makes a novel contribution by linking SCM typologies with ANN applications, complemented by an in-depth comparative LCA analysis. By outlining these contributions, this review clarifies its added value and offers insights into future advancements in the design of eco-friendly cementitious composites.

The novelty of this literature review lies in its systematic integration of LCA and ANNs within the context of SCMs, offering a comprehensive synthesis that has not been achieved in previous studies. While earlier reviews have often addressed either the environmental assessment of SCM-based cementitious composites or the predictive capabilities of data-driven models in isolation, this work uniquely bridges these domains by critically examining how ANN-based optimization can be directly coupled with LCA outcomes to achieve eco-efficient mix designs. By developing a critical analysis that links diverse SCM typologies with ANN applications across multiple case studies, this review not only consolidates fragmented research but also establishes a structured framework for evaluating the dual objectives of performance and sustainability. This dual focus advances the field by clarifying methodological inconsistencies, highlighting underexplored SCMs, and identifying opportunities where intelligent modeling can accelerate the adoption of greener binders. As such, this review contributes beyond the existing literature by transforming disparate findings into a coherent decision-support perspective, thereby strengthening the role of digital sustainability tools in guiding the design of next-generation cementitious composites.

The structure of the following sections of this manuscript is described below. Section 2 provides a detailed background to clarify essential concepts, including SCMs, LCA, and ANNs. In Section 3, the methodology employed to carry out this literature review is explained, i.e., the “Preferred Reporting Items for Systematic Reviews and Meta-Analyses (PRISMA)” guidelines. Section 4 presents in-depth analyses of each of the case studies found in the existing state-of-the-art that focus on LCA–ANN integration in SCM-based cementitious composite mix optimization. Subsequently, in Section 5, there is a comprehensive critical discussion on the current state of knowledge, highlighting key findings and emerging trends; comparing methodological approaches; examining theoretical perspectives; identifying gaps and limitations in the literature; addressing conflicting evidence and debates; and outlining the implications for practice, policy, and future research. Finally, Section 6 summarizes the main conclusions of this investigation.

## 2. Background

Presenting a clear background is essential to establish a common understanding of key concepts such as SCMs, LCA, and ANNs, which are fundamental to current research in eco-friendly cementitious composites. These concepts encompass interdisciplinary knowledge spanning the fields of materials science, environmental engineering, and computational methods. Without a concise explanation, readers may struggle to grasp the scope, relevance, and interconnection of the topics herein addressed. In this regard, a well-defined background ensures that the technical discussion that follows is coherent, accessible, and grounded in shared terminology.

### 2.1. SCMs

**Figure 2:**
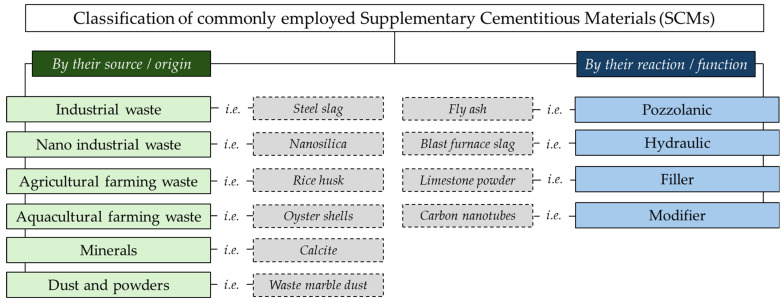
Classification of Supplementary Cementitious Materials. Adapted from [30].

The field of SCM is recognized as broad and continuously evolving, representing one of the principal research lines in the area of composite materials [29]. While several SCMs are well-established in the literature for their proven performance and compatibility with cementitious composites, ongoing studies continue to explore novel materials, leading to innovative approaches that expand the range of potential alternatives for construction sector. In this context, Figure 2 presents a classification of commonly utilized SCMs based on their source and function. As the concept indicates, SCMs are by-products that are typically used as substitutes for cement binders within composites. Particularly, in the case of concrete and mortar, they are most frequently employed as partial replacements for ordinary Portland cement (OPC), aiming to address both technological and environmental aspects [30].

From a technical perspective, the incorporation of SCMs aims to improve the key performance characteristics of cement-based materials, including fresh-state workability, mechanical strength, and long-term durability. Moreover, from an environmental point of view, the partial substitution of OPC with SCM positively contributes to reducing the carbon footprint of construction materials [31]. On the one hand, considering that cement production is responsible for high CO_2_ emissions during the calcination of limestone and high energy consumption during clinker production [32], the use of SCMs reduces the clinker in the mixtures, thereby reducing the carbon footprint of cementitious materials. 

On the other hand, SCM promotes the valorization of industrial, agricultural, and other process-derived residues that would otherwise be disposed of as waste [33]. In this regard, SCMs support both performance enhancement and implementation of sustainable, resource-efficient building practices.

### 2.2. LCA

**Figure 3:**
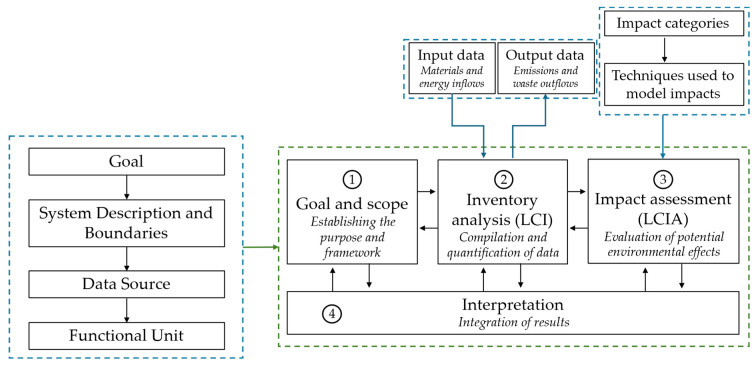
Framework for LCA model construction: from data acquisition to data interpretation. Adapted from [41].

LCA is the benchmark methodology for holistically quantifying the environmental impacts of products and processes [34,35,36]. This approach relies on a systematic framework standardized by ISO 14040 [37] and ISO 14044 [38], which evaluates the environmental burdens associated with all stages of a product’s life-cycle, from raw material extraction, processing, and manufacturing to distribution; use; and end-of-life treatment, including disposal or recycling [39,40]. These ISO standards define four main phases: (1) goal and scope definition, (2) Life-Cycle Inventory (LCI) analysis, (3) life-cycle impact assessment (LCIA), and (4) interpretation. These phases are interconnected, as illustrated in Figure 3.

The goal and scope definition phase is the initial and foundational step in any LCA study. It specifies the purpose of the assessment; establishes the functional unit, a quantitative reference that enables consistent comparisons across different systems; and delineates the system boundaries. In the context of cementitious materials, the functional unit is commonly defined as 1 ton (1 t) of cement or 1 cubic meter (1 m^3^) of concrete, providing a standardized basis for evaluating environmental impacts.

**Figure 4:**
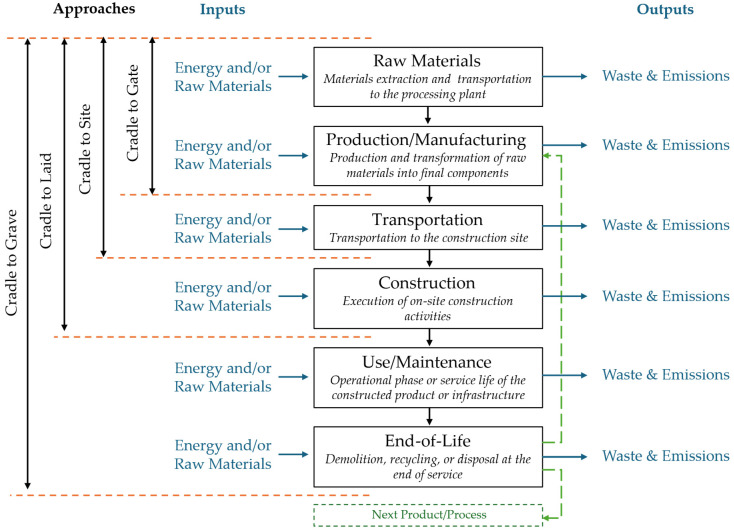
Life-cycle process flow. Adapted from [2].

The system boundaries determine which processes and life-cycle stages are included in the analysis. Two widely adopted approaches are (i) cradle-to-gate, which covers all stages from raw material extraction up to the point the product exits the manufacturing facility, and (ii) cradle-to-grave, which extends the assessment to include downstream processes such as transportation; construction (placement); use phase; and end-of-life scenarios including demolition, disposal, or recycling. These boundary frameworks are illustrated in Figure 4.

The LCI analysis phase involves systematic collection and quantification of all relevant inputs (e.g., energy, raw materials, and water) and outputs (e.g., emissions, solid waste, and wastewater) associated with each stage of the product’s life cycle. For cement incorporating SCMs, this stage requires particular attention to the origin, processing, and transportation of SCMs [8,29,42]. It is also essential to address the allocation of environmental burdens when dealing with industrial by-products such as fly ash or blast furnace slag, in accordance with ISO allocation principles [43,44,45].

At this point, the elementary flows characteristic of the cement systems under study are defined. Table 1 summarizes the typical input and output flows reported in the literature per ton of clinker, serving as a baseline for environmental modeling and comparison.

These inventory flows are translated into potential environmental impacts through characterization models, which quantify how specific emissions and resource uses contribute to various impact categories. Commonly used models include those for global warming potential (GWP), acidification, eutrophication, and resource depletion, among others [8,50,55]. For cementitious materials, the most relevant impact categories, based on frequency of use in the literature and regulatory frameworks, are summarized in Table 2**.** These categories serve as key indicators for evaluating and comparing the sustainability of different binder compositions and production routes.

Recent studies have reported reductions of up to 77% in CO_2_ emissions and 57% in embodied energy when replacing ordinary Portland cement with optimized blends of SCMs and recycled aggregates [8,29,42,56,57]. These results highlight the significant environmental potential of alternative cementitious systems when properly designed.

Finally, the interpretation phase integrates the outcomes from previous stages to support informed decision-making and identify opportunities for environmental improvement. This includes conducting sensitivity and uncertainty analyses, which are essential for validating the robustness of the conclusions. The interpretation phase plays a pivotal role in guiding the development of more sustainable construction materials and technologies [5,36,58].

### 2.3. ANNs

**Figure 5:**
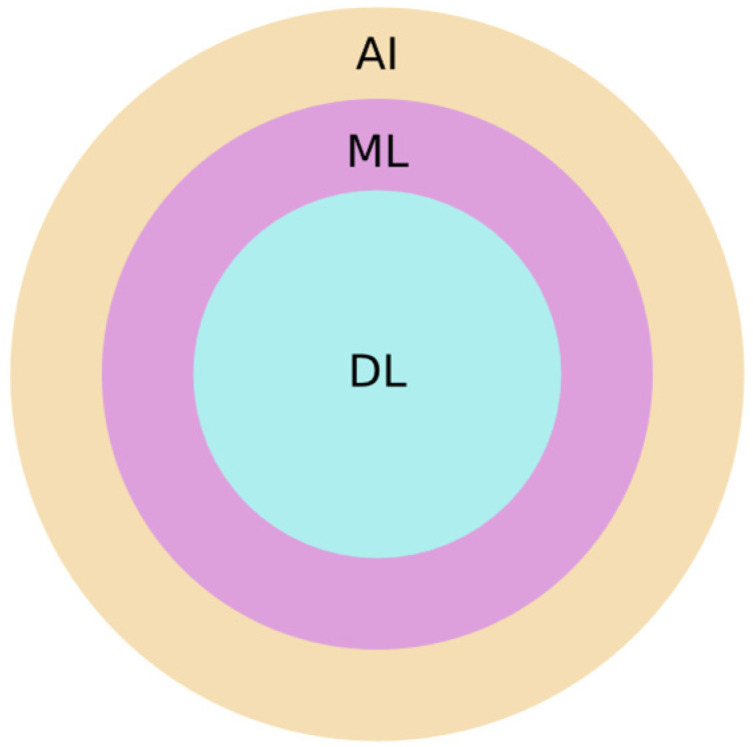
Hierarchical relationship between AI, ML, and DL. Adapted from [60,85].

Before focusing on ANNs, it is necessary to introduce more basic concepts, such as artificial intelligence (AI), machine learning (ML), and deep learning (DL). First of all, AI refers to the capability of machines/computers to imitate intelligent human behavior by performing tasks such as learning, reasoning, problem-solving, and perception [59,60]. AI encompasses several branches, including expert systems, natural language processing, robotics, computer vision, evolutionary computation, and machine learning (ML), which has become one of the most prominent [61,62]. ML is a branch of AI that enables systems to automatically learn patterns from data and improve their performance over time without being explicitly programmed [63,64]. Within ML, there are further sub-branches (e.g., supervised, unsupervised, reinforcement learning, and their mid-points) with their canonical algorithms [65,66]. On the one hand, there are regressions, support vector machines, decision trees, random forests, gradient boosting machines, K-nearest neighbors, and naive Bayes for supervised learning [67,68]. On the other hand, there are K-means clustering, hierarchical clustering, principal component analysis, independent component analysis, decomposing multivariate signals into additive subcomponents, and density-based spatial clustering of applications with noise, all of which are used for unsupervised learning [69,70]. Meanwhile, reinforcement learning encompasses algorithms such as Q-learning, state–action–reward–state–action, and policy gradient methods [71,72]. Another fundamental class of algorithms within ML, applicable across supervised, unsupervised, and reinforcement learning paradigms (as well as their hybrids), is known as ANNs [73,74]. Thus, DL emerged as a specialized subfield of ML centered on the use of ANNs [75,76]. Figure 5 illustrates the hierarchical relationship between AI, ML, and DL. In simple words, ANNs can be defined as a set of computational models designed to simulate the way biological neurons process and transmit information [77,78]. ANNs consist of layers of simple, interconnected processing units (the so-called neurons or nodes), where each unit transforms input data using weighted connections and activation functions [79,80]. Through iterative learning, ANNs adjust these weights to capture complex relationships within data, enabling them to perform tasks such as classification, prediction, and pattern recognition across various domains [81,82]. Their layered structure allows them to represent both linear and nonlinear mappings between inputs and outputs, making them highly versatile tools [83,84].

**Figure 6:**
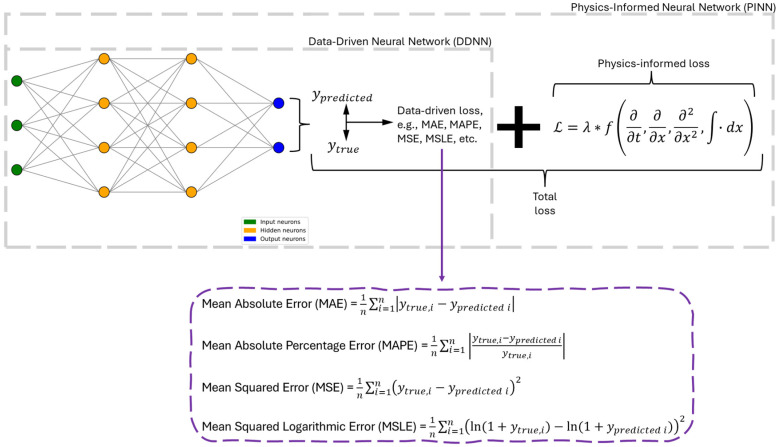
Comparative analysis of loss functions in DDNN and PINN. Adapted from [109,110].

**Figure 7:**
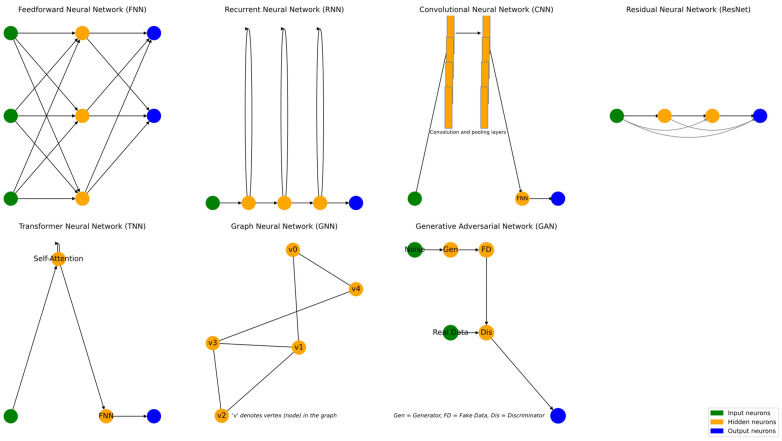
Simplified abstraction of information flows in the main types of ANNs. Adapted from: [111,112].

ANNs can be classified in two main ways: (i) based on how they compute the error during training, and (ii) based on their internal architecture, i.e., how information flows through the network. Figure 6 and Figure 7 depict those classification approaches. From the error calculation perspective, there are two broad types: the traditional Data-Driven Neural Networks (DDNNs) and the more contemporary Physics-Informed Neural Networks (PINNs) [86]. DDNNs rely solely on datasets and minimize a loss function derived from the difference between predicted and observed data without incorporating knowledge of the underlying physical systems [87,88]. In contrast, PINNs integrate physical laws, often expressed as partial differential equations, directly into the loss function to ensure that the network’s predictions obey known physical constraints, making them particularly valuable for modeling scientific and engineering problems with limited data [89,90,91]. Meanwhile, classification by architecture yields seven commonly recognized types of ANNs, each specialized for different types of data and tasks, namely Feedforward Neural Networks (FNNs), Recurrent Neural Networks (RNNs), Convolutional Neural Networks (CNNs), Residual Neural Networks (ResNets), Transformer Neural Networks (TNNs), Graph Neural Networks (GNNs), and Generative Adversarial Network (GANs) [92,93]. FNNs are the simplest type, where data flows unidirectionally from input to output through one or more hidden layers [94,95]. RNNs introduce loops within the architecture to allow memory of previous inputs, making them effective for sequential data [96,97]. CNNs apply filters to local regions of input data, enabling them to detect spatial hierarchies and patterns, which is ideal for image processing [98,99]. ResNets incorporate shortcut connections that allow gradients to bypass certain layers, which helps to train very deep networks without the vanishing gradient problem [100,101]. TNNs replace recurrence with self-attention mechanisms, enabling highly parallelizable models that excel in natural language processing [102,103]. GNNs generalize the neural network concept to graph-structured data, allowing nodes to aggregate information from their neighbors, and are used in domains such as social networks and molecular modeling [104,105]. Finally, GANs consist of a generator and a discriminator that are trained in opposition; the generator learns to produce realistic data while the discriminator learns to distinguish between real and fake data, making GANs powerful tools for text creation, image synthesis, data augmentation, and other generative tasks [106,107,108].

**Figure 8:**
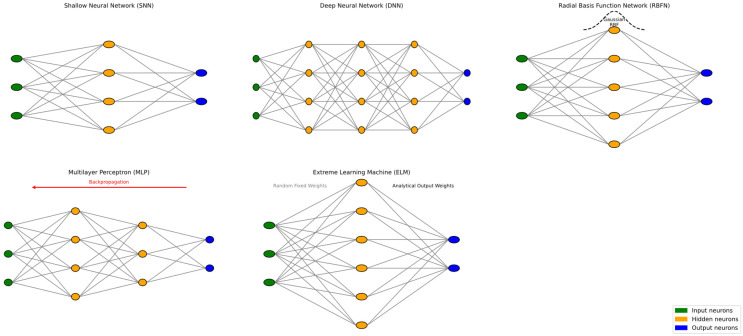
Simplified abstraction of the main subtypes of FNNs. Adapted from [135,136,137].

Although there are many subtypes of ANNs, it would be impractical to define them all, as this is not the objective of this literature review. Nevertheless, there are some of them that, due to their widespread use in the civil engineering sector, deserve mention. On the one hand, there are Thermodynamics-based Artificial Neural Networks (TANNs), a subset of PINNs that compute errors based on physical principles governed by the laws of thermodynamics, such as energy balance and entropy production [113,114]. On the other hand, it is also important to note that FNNs can be further categorized based on the complexity of their architecture, specifically in terms of the number of hidden layers and the activation functions employed [115]. Thus, it is possible to list at least five subtypes of FNNs (see Figure 8), namely Shallow Neural Networks (SNNs), Deep Neural Networks (DNNs), Radial Basis Function Networks (RBFNs), Multilayer Perceptrons (MLPs), and Extreme Learning Machines (ELMs) [116]. SNNs are composed of a single hidden layer that maps inputs to outputs using nonlinear activation functions [117,118]. DNNs extend this structure by stacking multiple hidden layers with densely connected neurons (i.e., all possible connections are established) to model complex hierarchical representations [41,119]. RBFNs use a hidden layer of neurons with radial basis activation functions, typically Gaussian, to approximate nonlinear functions based on distance from prototype centers [120,121]. MLPs refer to fully connected FNNs with one or more hidden layers, commonly trained using backpropagation [122,123]. Finally, ELMs are single-hidden-layer FNNs where the input weights are randomly assigned and fixed, and only the output weights are learned through a closed-form solution, enabling fast training [124,125].

It is essential to note that within the broader field of AI, including its ML-related subfields, there is no universally accepted consensus on terminology or classification frameworks. Even nowadays, there is a vigorous scientific debate about the precise definitions of AI, ML, DL, and ANNs. Consequently, the descriptions and conceptual distinctions presented herein should be understood as simplified abstractions intended to provide a clear and accessible overview of the subject matter. Figure 5, Figure 6, Figure 7 and Figure 8, in particular, are illustrative models designed for explanatory purposes; they do not capture the full depth or complexity of the underlying concepts. These topics remain subject to academic discussion and varying interpretations across the literature. Readers seeking a more comprehensive or critical perspective are encouraged to consult the following scholarly sources [62,126,127,128,129,130,131,132,133,134].

## 3. Methodology

**Figure 9:**
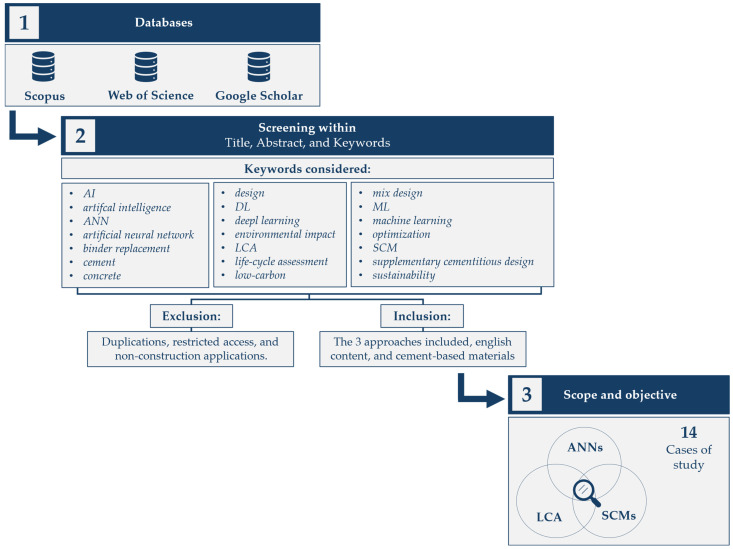
Workflow, scope, and objective of this literature review.

Figure 9 illustrates the workflow followed in the present research based on the selected scope and objectives of this literature review, which focuses on state-of-the-art research concerning cementitious composites incorporating SCMs designed through the integration of LCA and ANNs. The PRISMA methodology was employed [138,139], insofar as applicable, to guide the execution of this literature review. In this regard, a comprehensive search was conducted in the Scopus, Web of Science, and Google Scholar databases to identify academic sources that explore the intersection of SCMs, LCA, and ANNs for the design of cementitious composites. In order to capture the full scope of relevant studies, multiple combinations of keywords were used, both in abbreviated and expanded forms. These keywords included “AI”, “artificial intelligence”, “ANN”, “artificial neural network”, “binder replacement”, “cement”, “concrete”, “design”, “DL”, “deep learning”, “environmental impact”, “LCA”, “life-cycle assessment”, “low-carbon”, “mix design”, “ML”, “machine learning”, “optimization”, “SCM”, “supplementary cementitious materials”, and “sustainability”. This variety of search strings ensured the inclusion of studies with different terminologies and indexing styles, improving the comprehensiveness of the literature review.

Following a preliminary review of the records retrieved from database exploration, a screening process was conducted, resulting in the selection of only 14 research articles that directly address the topic under investigation (see Figure 9). These studies are [20,21,22,24,25,26,27,28,140,141,142,143,144]. Although the number of obtained articles may appear low, this is primarily due to the emerging nature of the topic, which lies at the intersection of multiple disciplines, including materials science, environmental engineering, and computational methods. It is essential to note that numerous other papers partially address the relevant topics; however, they do not fully align with the specific scope and objectives of this review and were, therefore, excluded from the final selection. For instance, de Paula Salgado et al. [145] examined the capabilities of various ML-based algorithms to optimize the incorporation of fly ash, limestone, and calcined clay as SCMs within a cradle-to-practical-completion LCA approach. Nonetheless, Paula Salgado et al. [145] did not include ANNs among the algorithms explored; hence, their case study falls beyond the scope of this literature review. Another interesting example is presented in the work of Tümay Ateş et al. [146] where the focus was on developing a sustainable cement-based binder rather than designing a cementitious composite, such as mortar or concrete, which places that investigation outside the defined objective of this review.

**Figure 10:**
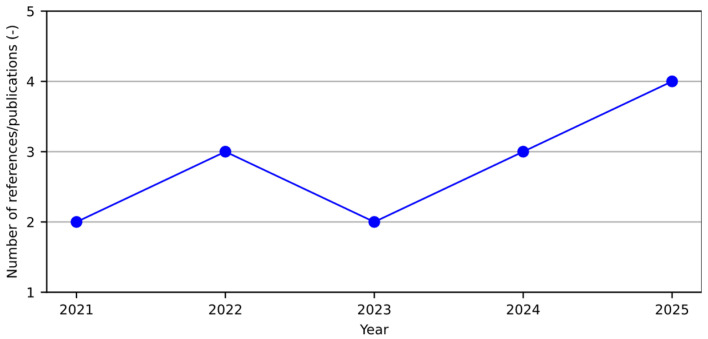
Growth pattern of academic outputs over time.

Although it is impractical to perform a comprehensive bibliographic analysis with just 14 research articles, a superficial inspection can still provide preliminary insights into the academic interest in the addressed topic. Figure 10, therefore, illustrates the temporal growth pattern of academic outputs related to the design of cementitious composites incorporating SCMs developed using an LCA–ANN integration framework. As shown in the graph, this line of research began in 2021 with two publications and has experienced a gradual increase, reaching four publications by mid-2025. It is essential to note that the literature review was conducted up to June 20 (2025), and additional articles may still be published later this year. The modest yet consistent rise in publications suggests a growing but still nascent interest in this interdisciplinary approach, reflecting an emerging recognition of sustainability-driven design tools in the construction materials field.

## 4. Research Case Studies

### 4.1. Overview of Case Study Approaches

In this section, a comprehensive analysis of multiple case studies is presented to illustrate the integration of LCA and ANNs for optimizing cementitious composites incorporating SCMs. The selected studies span a wide range of SCMs, such as fly ash, slag, silica fume, rice husk ash, and waste glass powder, and apply various ANN architectures, including multilayer perceptrons, convolutional models, and hybrid machine learning frameworks. Most investigations adopt a cradle-to-gate LCA perspective, with some extending to service life or hydration stages, and consistently report significant reductions in GWP, in some cases exceeding 70%. ANN models are employed to predict mechanical properties and environmental indicators, enabling data-driven mix design optimization with minimal experimental effort. This review of cases highlights how the LCA–ANN synergy supports informed decision-making in sustainable construction, demonstrating its versatility for reducing embodied emissions while maintaining or improving structural performance.

A comparative reading of the reviewed studies reveals important divergences in both the scope of LCA and the evaluation of ANN performance, which must be considered when interpreting the collective findings. Regarding LCA, most case studies adopt attributional cradle-to-gate boundaries [20,21,24], emphasizing impacts up to the production or mixing stage. While this ensures methodological consistency and reduces data uncertainty, it omits downstream phases such as transportation, service life, and end-of-life recycling. Notably, Xing et al. (2023) [144] partially extended boundaries to account for recycled aggregate scenarios, and Radwan et al. (2022) [142] included higher replacement levels within a cradle-to-grave context. These examples illustrate how boundary definitions and allocation choices may shift environmental comparisons, particularly when valorizing products. Hence, future work should pursue harmonization of LCA boundaries and explore broader system scopes to capture the full life cycle of SCM-based concretes.

On the ANN side, heterogeneity is equally pronounced. Several studies rely on straightforward feedforward or MLP architectures trained on experimental datasets [7,26,27], reporting accuracy through regression metrics such as R^2^, RMSE, or MAE. Others integrate metaheuristic or hybrid approaches, such as cuckoo optimization [7], teaching–learning-based optimization [21], or Taguchi–Grey Relational Analysis [25], to enhance predictive robustness and generate Pareto-optimal solutions. While most models achieved high predictive accuracy, differences in training data size, validation strategies, and reported performance metrics complicate direct cross-study comparisons. Standardized benchmarks for ANN evaluation, combined with transparent reporting of error metrics, would facilitate comparability and strengthen confidence in AI-driven mix design frameworks.

### 4.2. In-Depth Analyses of Key Studies

The following subsection presents a detailed overview of the 14 case studies identified through the literature review. Each investigation is described individually to highlight its key features, specific methodologies, and contributions to the field.

It is worth noting that this literature review distinguishes between explicit and implicit LCAs. On the one hand, explicit LCA refers to a structured and standardized analysis based on recognized methodologies (e.g., ISO 14040 and ISO 14044 guidelines [37,38]) including clearly defined system boundaries, functional units, and inventories. Conversely, implicit LCA involves general estimations of environmental impacts without strictly following formal LCA protocols, often using approximate data or simplified indicators.

#### 4.2.1. Case Study 1: Boukhelf et al., 2023

The study by Boukhelf et al. [24] focuses on mortars incorporating various binders, specifically exploring the environmental and mechanical impacts of using glass powder as an SCM. Four types of mortars are evaluated: those based on ordinary Portland cement or based on blast furnace cement, and either of these but partially replaced with glass powder at a 50% mass ratio. The LCA conducted is explicit, using an attributional approach with cradle-to-gate system boundaries. It comprehensively evaluates raw material inputs and energy demands, highlighting substantial reductions in environmental impacts due to the inclusion of glass powder. The ANN employed is an MLP regressor, which uses the heat hydration test as the sole input to predict the different hydration modes of binders. The LCA and ANN were integrated to assess and optimize binder formulations, enabling the classification of real-time hydration modes with minimal experimental inputs. The research concludes that substituting traditional Portland cement with glass powder effectively reduces environmental impacts, and the ANN model provides a practical and rapid tool for predicting hydration behavior. The combined LCA–ANN framework advances sustainable mix design by enabling efficient screening of eco-friendly binders.

#### 4.2.2. Case Study 2: Faridmehr et al., 2021

The investigation by Faridmehr et al. [7] examines several SCMs (i.e., ground-granulated blast furnace slag, fly ash, palm oil fly ash, and waste ceramic powder) for producing both mortars and concretes. These materials were mixed in varying proportions, and each of these SCMs was characterized in terms of chemical and physical properties. The LCA performed was explicit, adopting a cradle-to-gate system boundary that was extended to include mechanical and durability performance, thus incorporating service life considerations. The functional unit was defined as per cubic meter of cementitious material, and CO_2_ emissions, along with embodied energy, were the primary environmental impact categories. An FNN combined with a metaheuristic algorithm was assembled using eight input parameters, including SCM proportions, oxide ratios, and age, to predict CO_2_ emissions and embodied energy as output variables. The ANN was optimized using the cuckoo optimization algorithm, and its final weights and biases were utilized to design mixes that targeted specific environmental and mechanical outcomes. This integration of LCA with ANN modeling enabled the identification of optimized compositions with reduced environmental burden and enhanced durability, demonstrating the potential for intelligent mix design that reduces carbon footprint while ensuring structural integrity.

#### 4.2.3. Case Study 3: Miao et al., 2025

The research by Miao et al. [22] explores the design and optimization of concrete incorporating waste glass powder as an SCM. The waste glass powder is used as a partial replacement for ordinary Portland cement, with replacement dosages reaching up to 50% by mass of cementitious material. This investigation performs an implicit LCA using an attributional approach, adopting (virtually) cradle-to-gate system boundaries with a functional unit of 1±0.03 m^3^ of concrete. Environmental indicators assessed include abiotic depletion, GWP, ozone depletion potential, acidification potential, eutrophication potential, and photochemical ozone creation potential. This study combined a backpropagation neural network, a GAN, and other AI-based algorithms to optimize the mix design. The input variables comprised the dosage of materials, particle sizes, and curing age, while the output variables were slump, compressive strength, and total charge passed. The LCA–ANN integration was achieved through a multi-objective optimization framework to generate optimal mix designs that balance mechanical performance, durability, environmental impact, and even production cost. The study concludes that glass powder concrete mixtures with up to 50% cement replacement may deliver comparable strength and significantly enhanced durability while reducing CO_2_ emissions and costs.

#### 4.2.4. Case Study 4: Mungle et al., 2024

The case study by Mungle et al. [20] analyzes concrete mixtures as the addressed cementitious composite, aiming to enhance its strength while reducing environmental impacts. It incorporates various SCMs, including fly ash, slag, silica fume, and metakaolin, with replacement dosages ranging from 10% to 50% by weight of the mix. The research explicitly employs LCA using an attributional approach with (practically) cradle-to-gate boundaries; it considers GWP (in terms of equivalent CO_2_ emissions) as the primary impact category, with a functional unit based on 1 m^3^ of concrete. A hybrid ANN system is adopted, combining gradient boosting machines and CNNs for microstructural image analysis. The input variables included material ratios, curing conditions, and image features, and the output variables focused on predicting compressive strength. The integration of LCA and ANN is achieved through multi-objective optimization, balancing strength, cost, and environmental performance by coupling predictive outputs with sustainability-related impact functions. This LCA–ANN framework led to concrete mixtures achieving over 40 MPa of compressive strength with 20% CO_2_ emission reduction and less than 10% cost increase, highlighting a significant advancement in sustainable concrete design through synergistic SCM optimization and intelligent data-driven modeling.

#### 4.2.5. Case Study 5: Nasrollahpour et al., 2024

The study by Nasrollahpour et al. (2024) [21] presents a comprehensive approach to optimizing alkali-activated concretes incorporating various SCMs, such as fly ash, slag, and rice husk ash. The research integrates a cradle-to-gate LCA with an ANN optimization to minimize environmental impacts while maintaining mechanical performance. The functional unit is defined as 1 m^3^ of concrete, and key impact categories assessed include GWP, abiotic resource depletion, and energy consumption.

An FNN is trained using 91 experimental mix designs, incorporating 12 input variables (e.g., binder type, SCM dosage, Na_2_O and SiO_2_ molar ratios, and curing conditions) and 4 outputs: compressive strength, CO_2_ emissions, total energy use, and cumulative cost. The ANN is embedded within a multi-objective optimization framework using the Teaching–Learning-Based Optimization (TLBO) algorithm to generate Pareto-optimal solutions balancing environmental and structural performance. The findings show that mixes incorporating rice husk ash and slag can reduce CO_2_ emissions by up to 53% and energy use by 48%, while achieving target compressive strengths ≥ 35 MPa. This study exemplifies the synergy between ANN-based predictive modeling and LCA for sustainable concrete design. The combined framework facilitates data-driven selection of optimal binder combinations, enabling both low-carbon construction and performance reliability in SCM-based concretes.

#### 4.2.6. Case Study 6: Onyelowe et al., 2022a

The study by Onyelowe et al. (2022) [140] explores a data-driven optimization framework for low-carbon geopolymer concrete incorporating several SCMs, including fly ash, granulated blast furnace slag (GBFS), and waste glass powder. The researchers employed an integrated methodology combining LCA and ANNs to evaluate and optimize the environmental and structural performance of the mixes.

The LCA was conducted following a cradle-to-gate approach. Impact categories included GWP, acidification potential, and embodied energy. The functional unit was 1 m^3^ of geopolymer concrete. An FNN model with backpropagation was trained on 60 experimental datasets using inputs such as SCM proportions, curing duration, alkaline activator concentration, and binder ratio. The outputs modeled included compressive strength, CO_2_ emissions, and energy demand.

Optimization was achieved via multi-objective sensitivity analysis, highlighting optimal SCM blends (notably fly ash + GBFS) that reduced CO_2_ emissions by up to 61% compared to OPC-based mixes, while delivering compressive strengths exceeding 30 MPa. Waste glass powder also showed promise in reducing environmental loads with minimal compromise on mechanical performance. This study validates the application of ANN–LCA coupling as an efficient decision-support tool in green concrete design. It provides a robust framework for predicting performance outcomes and minimizing environmental burdens associated with SCM-rich geopolymer systems.

#### 4.2.7. Case Study 7: Onyelowe et al., 2022b

The work by Onyelowe et al. (2022) [141] investigates a sustainable alternative to Portland cement using alkali-activated binders that incorporate fly ash, slag, and cement kiln dust (CKD). The study adopts a dual approach by combining an ANN-based predictive model with a cradle-to-gate LCA to evaluate both environmental and mechanical performance of blended geopolymer concretes.

The ANN model was trained using 81 datasets, with 9 input parameters including binder ratios, SCM percentages, molarity of sodium hydroxide, and curing conditions. Output parameters were compressive strength, CO_2_ emissions, and embodied energy. The architecture used was an MLP with backpropagation, optimized for minimizing prediction error and maximizing generalization.

For the LCA, the ISO 14040-compliant framework assessed impacts such as GWP, cumulative energy demand, and acidification. The functional unit was 1 m^3^ of concrete. The results showed that replacing OPC with SCMs (notably CKD and slag) led to reductions of 45–65% in GWP and significant declines in energy use, while achieving compressive strengths above 30 MPa. The study demonstrates the effectiveness of the integrated ANN–LCA approach for optimizing geopolymer concrete compositions. It provides a scalable methodology to balance sustainability metrics with engineering performance, emphasizing the viability of industrial by-products in low-carbon construction.

#### 4.2.8. Case Study 8: Onyelowe et al., 2022c

In this study, Onyelowe et al. (2022) [28] present an integrated framework for optimizing geopolymer concrete mixes incorporating rice husk ash (RHA), fly ash, and GBFS as SCMs. The research is aimed at developing eco-efficient concrete mixtures by combining LCA with ANN modeling and optimization algorithms.

The LCA is conducted with cradle-to-gate boundaries and uses a functional unit of 1 m^3^ of concrete. Environmental indicators include GWP, cumulative energy demand, and acidification. The LCA results show that replacing OPC with a ternary blend of RHA, fly ash, and GBFS can reduce GWP by up to 59%, depending on mix design and curing conditions.

An MLP is used to model the compressive strength and environmental impacts of 75 different mix formulations. The model incorporates variables such as SCM percentages, activator molarity, binder ratios, and curing regimes. Optimization is conducted using a hybrid Artificial Bee Colony (ABC) algorithm, producing Pareto-optimal solutions that balance environmental performance and mechanical strength. The study concludes that geopolymer concrete with ternary SCM blends can achieve compressive strengths >35 MPa while significantly reducing environmental impacts. The integrated ANN–LCA approach proves effective for designing sustainable concrete systems with reduced reliance on Portland cement and enhanced valorization of agricultural and industrial wastes.

#### 4.2.9. Case Study 9: Padavala et al., 2024

The research conducted by Padavala et al. (2024) [27] proposes an advanced framework for optimizing concrete mixes incorporating fly ash and silica fume as SCMs, aiming to reduce environmental impact while preserving high mechanical performance. The methodology integrates a comprehensive LCA with predictive ANN modeling and parametric analysis, supporting data-driven decision-making in green concrete design.

The LCA is conducted under ISO 14044 standards, with cradle-to-gate boundaries and a functional unit of 1 m^3^ of concrete. Key impact categories include GWP, abiotic resource depletion, and embodied energy. The incorporation of SCMs such as fly ash and silica fume led to a reduction of up to 63% in CO_2_ emissions, relative to traditional OPC-based concretes.

The predictive ANN model is based on an MLP architecture with backpropagation, trained using experimental datasets comprising mix proportions, water/binder ratios, and curing regimes. Outputs include compressive strength, split tensile strength, and environmental indicators derived from the LCA. The study also performs sensitivity analysis to identify the influence of individual variables on both performance and sustainability. Through the integration of ANN predictions and LCA outputs, the authors identify optimal mix designs that achieve compressive strengths over 40 MPa with significant environmental savings. The research highlights how ANN–LCA coupling can facilitate the development of low-carbon, high-performance concretes through the targeted use of SCMs.

#### 4.2.10. Case Study 10: Radwan et al., 2022

The research work by Radwan et al. [142] examines the impact of replacing ordinary Portland cement with SCM, specifically GGBFS and fly ash (FA), to improve both the strength and environmental performance of the mixtures employed. The study considered high replacement of OPC, specifically 50% and 70% by weight, along with different types of SCM, including two sources of coal fly ash (FA-I and FA-II) and GGBS. This study explicitly employs LCA, specifically a cradle-to-gate LCA analysis, to evaluate the environmental performance of OPC blends production. It considers GWP as the primary impact indicator, as it accounts for the highest impact contributed by cement blend production. The functional unit for the LCA was based on one cubic meter of OPC mixture. 

In addition, an FNN was adopted to predict the properties of the OPC blends, particularly their eco-mechanical performance. The input variables for the ANN included the contents of OPC, GGBS, and fly ash. In contrast, the output variables focused on predicting the eco-mechanical index, defined as the GWP per unit of 90-day compressive strength (GWP/strength ratio). The integration of LCA and ANN is achieved through the development of this prediction model for the eco-mechanical index.

The results obtained from the integrated LCA–ANN framework led to the following key findings. Ternary OPC–GGBS–fly ash blends outperform binary blends when appropriate SCM combinations were adopted. Fly ash improved workability due to its morphology, compensating for the reduced flow associated with GGBS. Replacing OPC at high volumes reduced early-age strength, particularly in OPC–FA blends, but ternary mixtures with GGBS recovered strength at later ages. Blends with 50% OPC replacement and 20–30% fly ash exhibited both optimal flow and strength performance. Moreover, from an environmental standpoint, the control mixture yielded the highest impact, with GWP values of 574 kg CO_2_-eq/m^3^. In contrast, 50% and 70% OPC replacement reduced GWP to approximately 380 and 273 kg CO_2_-eq/m^3^, respectively. The 70% ternary blends with 10–20% fly ash showed the lowest GWP/strength ratio, achieving nearly 60% impact reduction per unit strength. The ANN model reliably estimated compressive strength, reaching a low mean square error (MSE) of 0.0997, which validates its use as a predictive tool. Hence, the authors recommend the OPC–GGBS–fly ash ternary blends, with a fly ash content limited to 20%, to meet enhanced mechanical and environmental performance.

#### 4.2.11. Case Study 11: Rahman and Lu, 2024

The case study presented by Rahman and Lu [143] introduces *EcoBlendNet*, a PINN developed to minimize the use of SCM in cement blends by estimating strength development and carbon emissions during hydration. In contrast to conventional finite element or machine learning-based models, *EcoBlendNet* incorporates experimental data and chemophysical concepts of cement hydration, allowing for accurate, mesh-free analysis with limited training data (as low as 5%). 

For this work, three different mixtures were considered for the calibration of the tool: moderate-heat Portland cement (MPC300), cement–fly ash (FA45), and cement–slag (SG80), allowing *EcoBlendNet* to estimate key properties during the hydration process, such as maturity, heat evolution, and strength development. Additionally, an implicit LCA was conducted in this research, evaluating the environmental assessment of cement hydration, specifically focused on energy consumption and CO_2_ emissions, addressing water consumption and heat generation. The results indicated that SCM blends exhibit lower early-age temperature peaks compared to conventional cement but achieve equivalent compressive strengths after seven days. SCMs were also found to promote hydration with increased capillary water availability and better reactant diffusion.

About environmental context, SCM use resulted in considerable emission reductions through the incorporation of fly ash and slag, with FA45 and SG80 blends attaining average emission reductions of 36% and 63% for CO_2_ and 33% and 73% for chemically bound water, respectively, corresponding to emission reductions up to 700 kg CO_2_-eq/m^3^ and 950 kg CO_2_-eq/m^3^ for fly ash and slag-based mixtures. These outcomes highlight the model’s potential in estimating the eco-efficiency of SCMs while facilitating circular economy applications through by-product valorization. Overall, *EcoBlendNet* presents a high-performance predictive tool for sustainable concrete composition and is suitable for informing material optimization while ensuring physical interpretability and accuracy.

#### 4.2.12. Case Study 12: Rizwan et al., 2025

The research conducted by Rizwan et al. [26] explores the potential of sugarcane bagasse ash (SCBA), a by-product of the sugar industry’s cogeneration process, as a sustainable SCM in concrete. This study focuses on optimizing compressive strength and evaluating environmental performance through an explicit LCA procedure. Thus, 4 ML-based models were introduced, namely an ANN, a random tree, a random forest, and gradient boosting machine. For this purpose, the addressed database contained 2616 entries, including variables such as cement, SCBA, fly ash content, water-to-binder ratio, aggregates, and superplasticizer.

Of all the analyzed models, the gradient boosting machine exhibited the optimum predictive capability, facilitated by Shapley Additive Explanation (SHAP) values, which highlighted the water-to-binder ratio and cement content as having a decisive influence. The findings observed in the optimized mixtures exhibited a strength of as high as 40 MPa using lower water–binder ratios (0.42–0.48) and a minimum use of cement, indicating that SCBA has potential for matrix density enhancement at moderate use ratios. 

Moreover, the LCA, particularly conducted on a cradle-to-gate basis in this research, estimated the GWP and other emissions (i.e., SO_2_, CO, PM_1__0_, and NO_x_), indicating a significant reduction in environmental impacts compared to conventional concrete, with a notable sensitivity to the water–binder ratio in particular. SCBA results from carbon-neutral production and addresses local waste disposal issues; hence, its use would contribute to emission reduction and support circular economy practices. Therefore, SCBA concrete is proposed by the authors as a sustainable and technically viable alternative that offers environmental benefits, as well as performance optimization and emission reduction, on a sustainable construction path.

#### 4.2.13. Case Study 13: Siddiq et al., 2025

The study proposed by Siddiq et al. [25] focuses on the development of fly-ash-based geopolymer concrete (FA-GPC) as a sustainable alternative to conventional cement-based systems, aiming to optimize both mechanical performance and environmental outcomes. The study is conducted using an integrated methodology that combines Taguchi–Grey Relational Analysis (GRA) and ANN, trained on a dataset compiled from 83 studies comprising over 1000 data values, including fly ash content, NaOH/Na_2_SiO_3_ ratio, and curing conditions. Additionally, a cradle-to-gate explicit LCA was conducted to quantify environmental performance.

The optimized FA-GPC formulation achieved a compressive strength of 90.9 MPa alongside a CO_2_ emission reduction of 78%, decreasing from 252.09 to 55.0 kg/m^3^. The ANN model demonstrated high predictive capability for strength and emissions, with R^2^ values exceeding 0.95, confirming its robustness in estimating eco-mechanical performance. In terms of the LCA, which accounted for emissions from raw material extraction, transportation, and curing processes, the results projected an annual reduction of approximately 3,941 tons of CO_2_ for construction projects utilizing 1,000 m^3^ of FA-GPC.

Moreover, Grey Relational Analysis revealed that molarity, heat-cure temperature, SiO_2_ content, and coarse aggregate had positive impacts on workability and compressive strength. In contrast, increased concentrations of NaOH, Na_2_SiO_3_, and Na/Al ratios were unfavorable. In general, the research validates the suitability of FA-GPC as a high-performance and low-pollution binder, thereby justifying industrial waste valorization, emission reduction, and measures promoting a circular economy. The hybrid optimization method proposed here presents a data-driven route to sustainable mix design for concrete.

#### 4.2.14. Case Study 14: Xing et al., 2023

In this investigation, Xing et al. [144] evaluates different experimental mix designs extracted from multiple scientific studies in the literature, to conduct a comprehensive analysis of their environmental behavior. The studies evaluate the environmental performance of sustainable concrete mixtures with recycled aggregates (RA) and SCMs, specifically fly ash, slag, and silica fume, to reduce the carbon footprint of traditional concrete production. Employing a database of over 500 concrete mix designs compiled from the literature, the researchers established an explicit LCA, based on the cradle-to-gate approach under Australian conditions, encompassing the end-of-life scenarios for RA.

LCA results indicated that the incorporation of RA consistently reduced environmental burdens, such as GWP and fossil fuel utilization, primarily due to avoided landfilling and reduced transportation. Moreover, additional findings demonstrated that SCMs were more effective than RA in reducing environmental impacts within a broader range of indicators, including acidification, eutrophization, human toxicity, and ecotoxicity. 

The dual use of RA and SCMs enhanced environmental performance and mechanical concrete properties, offsetting potential performance losses due to RA. It also examined the sensitivities of environmental impacts to SCM allocation scenarios, showing that assuming SCMs were wastes (zero allocation) yielded the maximum environmental benefits. In contrast, full allocation had the potential to counteract these perceived benefits, particularly for silica fume, due to the energy-intensive production of ferrosilicon. Economic allocation is the most recommended approach, yet it is subject to market variability. In addition, this study examined the contribution of alternative fuels in cement production by benchmarking German practices against Australian practices. Germany’s high substitution rate of alternative fuels (approximately 70%) resulted in remarkable emissions reductions and offered a potential strategy for carbon emissions reductions. 

An MLP had also been developed to predict environmental impacts based on technical mix parameters; however, improvement beyond that, e.g., individual SCMs, was recommended to enhance the model’s accuracy. Overall, this research outlines a robust platform for optimal sustainable concrete design by integrating LCA and ML models.

## 5. Discussion

The integration of LCA methodology and ANN approaches for optimizing cementitious composites incorporating SCMs constitutes a significant and recent research direction in the field of sustainable building materials. As explained in the previous sections, this combined methodological framework enables a systematic evaluation of both environmental and mechanical indicators’ performance, hence contributing to supporting progress in two critical and pursued areas of the construction materials sector: (i) improvement of technical performance and (ii) reduction in the environmental footprint.

In this line, this section provides an objective synthesis and evaluation of the evidence collected from the fourteen selected case studies. Through comparative analysis, it identifies convergences and divergences across methodologies, highlighting current limitations in the literature, and explores trends and gaps. In addition, the practical implications of the findings and essential directions for future research are discussed.

### 5.1. Summary of Key Findings

In order to facilitate a structured discussion, Table 3 summarizes key features of each case study, including the types of SCMs used, the composite considered for the study, ANN configurations, GWP reductions, compressive strength performance, and LCA scope. This overview highlights the methodological diversity and practical relevance of LCA/ANN frameworks in advancing sustainable and intelligent mix design for low-carbon construction materials.

The systematic review of the fourteen selected case studies shows the growing importance of integrating LCA and ANN when using SCM, with the aim of optimizing the composites. In this line, a recurring finding observed in the case studies is the potential of bringing these methodologies to improve mechanical strength and reduce carbon footprint simultaneously, specifically GWP. For instance, ternary and binary blends, particularly those incorporating fly ash, slag, or agricultural by-products (i.e., sugar cane bagasse ash), displayed consistently enhanced “Eco-mechanical” indices when compared to conventional cement mixtures. Moreover, the reviewed investigations highlight that predictive models exhibit high reliability in estimating both compressive strength and emission metrics, even in the presence of a limited training dataset. 

In parallel, LCA applications reveal significant reductions in terms of carbon emissions when increasing the replacement levels of OPC. It is worth noting that functional units normalized to mechanical performance (i.e., MPa) are gaining interest as established and accurate indicators of sustainability. Furthermore, in order to enhance the interpretability and resilience of decision-making, hybrid frameworks are increasingly using multi-criteria optimization techniques (i.e., Grey Relational Analysis) or explainability tools (e.g., SHAP values). One of the more significant findings to emerge from this work is the technical viability and environmental effectiveness of SCM in cement-based materials revealed by the LCA–ANN framework, underscoring the potential of this integrated methodology to guide the research into the sustainable material development required in the construction sector.

### 5.2. Comparative Analysis of Methodologies

The reviewed studies employ diverse methodologies to integrate LCA and ANN for optimizing SCM inclusion in cement composites. Most adopt an attributional cradle-to-gate LCA framework, though system boundaries vary, with some extending to durability (Faridmehr et al., 2021) [7] or end-of-life scenarios (Xing et al., 2023) [144]. ANN architectures also differ, ranging from MLP (Boukhelf et al., 2023) [24] to hybrid models combining gradient boosting and CNNs (Mungle et al., 2024) [20]. Predictive inputs commonly include mix design proportions, curing conditions, and material properties, while outputs focus on mechanical performance and environmental indicators. A key distinction lies in optimization techniques: some studies use metaheuristic algorithms like cuckoo optimization (Faridmehr et al., 2021) [7] or artificial bee colony (Onyelowe et al., 2022c) [28], whereas others leverage multi-objective frameworks (Miao et al., 2025) [22]. The various ways of combining these methodologies yield different results and performances, which are summarized in Table 4. Despite variations, all methodologies converge on a shared goal, i.e., balancing sustainability and performance through data-driven mix design.

### 5.3. Theoretical Perspectives and Frameworks

The studies are grounded in two complementary theoretical frameworks: environmental systems analysis and ML-based predictive modeling. LCA provides a structured approach to quantify impacts, typically focusing on GWP and energy-related quantities (e.g., embodied energy), though some expand to broader indicators like acidification (Xing et al., 2023) [144]. ANN models, often informed by physicochemical principles (Rahman et al., 2024) [143], serve as surrogate models to bypass costly experimentation. Theoretical integration occurs through multi-objective optimization, where Pareto fronts reconcile conflicting goals (e.g., strength vs. CO_2_ emissions). Notably, several studies embed circular economy principles, valorizing industrial by-products (e.g., fly ash and slag) as SCMs. Frameworks like *EcoBlendNet* by (Rahman et al., 2024) [143] further enhance interpretability by incorporating domain knowledge, bridging the gap between empirical data and theoretical hydration mechanics.

### 5.4. Gaps and Limitations in the Existing Literature

While the authors recognize that ANN are capable of solving complex problems, including highly nonlinear problems by using interconnected computing elements [147], in addition to the rising adoption observed in LCA–ANN models for the optimization of SCM-based cementitious composites, several drawbacks persist in the current literature. Among the common disadvantages is the lack of standard datasets and unified indicators of performance, which impedes precise cross-comparisons between different studies, thereby reducing the reproducibility of model outputs. Moreover, in many cases, the ANN models are aimed towards specific given experimental conditions with limited external verification, raising concerns among users of these methodologies. 

Likewise, applications of LCA vary considerably in terms of the scope, inventory data, allocation methods, and impact categories applied. While the cradle-to-gate approach dominates the studies, the absence of standard functional units, in addition to geographical harmonization, further attenuates the comparability. Furthermore, the lack of data regarding both region-specific SCM sources and end-of-life scenarios remains a significant impediment in terms of a robust simulation of environmental repercussions.

Another gap includes the null or limited integration of two essential columns in the field of cement-based materials: (i) long-term durability parameters and (ii) service life performance [148], both within ANN models and LCA assessments. Most studies are focused on the short-term or the early age of mechanical properties, while disregarding crucial parameters such as permeability, shrinkage, or resistance to carbonation, which have a significant influence on environmental performance over the long term. Additionally, whereas several case studies utilize hybrid SCM systems (e.g., ternary blends), relatively few include comprehensive sensitivity or uncertainty analysis to establish robustness of prediction. To conclude this section, ethical and practical considerations (i.e., economic feasibility, resource accessibility, or social acceptability) are rarely addressed, serving as a barrier to the transferability of results to real-life decision-making, a crucial attribute in the academic field.

### 5.5. Trends and Emerging Themes

The detailed revision of the selected case studies reveals a persistent trend towards integrating LCA and ANN techniques as an implementation strategy for optimizing cementitious materials by incorporating SCMs. Here, a noticeable trend away from single-objective analysis (predominantly centered on strength or CO_2_ emissions) can be observed towards multi-objective systems, which are simultaneously capable of evaluating these two aspects while incorporating additional variables (such as cement hydration, eutrophication, and acidification). Here, the trend shows and reinforces the awareness of the highly complex interdependencies that define the nature of sustainable material design, becoming critically relevant when applied to the sector of cement-based materials, given the wide variety and dispersity commonly found within their properties. Such heterogeneity is recognized as an inherent characteristic of these materials, underscoring the need for robust, integrative prediction and modeling techniques.

Furthermore, new trends suggest a shift away from purely empirical and data-driven methods to hybridized approaches. Growing, workability, longevity, and long-term behavior are increasingly incorporated into ANN models as part of the effort to capture real-world applications better. The use of regionalized LCA inventories and context-adaptive SCM availability also speaks to a trend towards local context and condition-based assessments of sustainability. At the same time, the use of more complex artificial intelligence approaches and feature selection techniques is emerging as a way to add the ability to provide better transparency and interpretation of the predictive models. Overall, these trends indicate a shift towards comprehensive, adaptive, and context-aware approaches, which align with the objectives of the broader circular economy and the strategy of decarbonization.

### 5.6. Conflicting Evidence and Debates

While the studies collectively advocate for SCMs, debates persist regarding optimal replacement levels and material combinations. For instance, Boukhelf et al. (2023) [24] report successful 50% glass powder substitution, whereas Radwan et al. (2022) [143] caution against early strength loss in high-volume fly ash mixes. Discrepancies also arise in environmental impact allocation; for instance, Xing et al. (2023) [144] highlight how assumptions (e.g., waste vs. economic allocation for SCMs) significantly alter LCA results. Additionally, the efficacy of ANNs depends on dataset quality; Rizwan et al. (2025) [26] note that gradient boosting outperforms ANNs for certain predictions, suggesting no universal “best” model. Geopolymer systems (Onyelowe et al., 2022a) [140] face scrutiny over long-term durability and scalability, with some studies (Siddiq et al., 2025) [25] reporting exceptional performance but others questioning industrial feasibility. These conflicts underscore the need for standardized benchmarking and real-world validation.

### 5.7. Implications for Practice and Policy

This literature review underscores actionable pathways for decarbonizing construction. Basically, ANN–LCA integration enables rapid screening of low-carbon mix designs, with studies like Padavala et al. (2024) [27] demonstrating 40 MPa concretes achieving 20% CO_2_ reductions. Policy-wise, findings support incentivizing SCM use (e.g., tax breaks for fly ash adoption) and mandating LCA-based procurement criteria. Nevertheless, barriers remain, including inconsistent SCM availability and the need for training in data-driven design. Radwan et al. (2022) [143] advocate for ternary blends (OPC–slag–fly ash) as a pragmatic compromise, while Rahman et al. (2024) [143] emphasize tools like *EcoBlendNet* to guide specifications. For circular economy alignment, policies must address allocation ambiguities (Xing et al., 2023) [144] and promote industrial symbiosis, ensuring waste-derived SCMs are prioritized. Collectively, these investigations provide a blueprint for transitioning to the production of eco-friendly cementitious composites, marrying technical innovation with regulatory foresight.

In order to accelerate the adoption of LCA–ANN-driven optimization for the design of sustainable cementitious composites with SCM inclusion, policymakers are strongly advised to implement the following measures:

- Mandate LCA-based environmental declarations: Require construction material producers to disclose carbon footprints and environmental impacts using standardized cradle-to-gate LCA methodologies.
- Update building codes for SCM inclusion: Revise regulations to allow higher SCM replacement ratios in concrete mixes, provided ANN-validated performance meets structural requirements.
- Fund AI-driven material research: Establish grants and public–private partnerships to develop ANN models for predicting SCM performance, focusing on industrial by-products like fly ash, slag, and recycled glass.
- Introduce carbon pricing or tax incentives: Offer tax breaks or subsidies for manufacturers using LCA–ANN-optimized low-carbon mixes, penalizing high-emission alternatives.
- Create open data repositories: Support centralized databases of material properties and LCA results to improve ANN training and global benchmarking.
- Promote industry-academia collaboration: Encourage joint initiatives between researchers, tech firms, and construction companies to scale AI-based mix design tools.
- Standardize LCA allocation methods: Develop clear guidelines for environmental impact accounting, particularly for waste-derived SCMs, to ensure consistency in sustainability claims.
- Incentivize pilot projects: Fund real-world demonstrations of LCA–ANN-optimized concretes to prove feasibility and encourage market adoption.

While the reviewed studies collectively demonstrate the potential of ANN–LCA frameworks for sustainable concrete design, several practical challenges remain. First, industrial implementation is constrained by the need for specialized expertise, high computational demand, and integration with existing workflows, which limit adoption in practice [146]. Second, the accuracy of ANN predictions is often affected by data limitations; most studies rely on relatively small or heterogeneous datasets, raising concerns about generalizability and robustness across diverse conditions [115]. Third, regional variability in SCM sourcing strongly influences LCA outcomes, as transportation distances, energy mixes, and allocation assumptions can significantly alter environmental benefits [43,144]. Addressing these barriers will require methodological innovation, standardized datasets, and collaborative efforts to contextualize results for specific geographical and industrial settings.

### 5.8. Recommendations for Future Research

Future research should prioritize the creation of harmonized and comprehensive datasets that integrate durability-related properties into the mechanical and environmental performance of cementitious composites after the incorporation of SCMs, considering long-term durability as one of the most persistent issues in the field [149]. Moreover, expanding the LCA boundaries (e.g., cradle-to-grave) and including additional categories can provide a more comprehensive understanding of SCM. In addition, an essential parameter for ensuring comparability in these frameworks remains in addressing allocation issues through harmonized and standardized methodologies. 

Regarding the modelling approach, while the authors acknowledge the complexity represented by this issue to be addressed, further research could enhance advanced hybrid models trained on established, proven datasets. Additionally, based on both the methodologies and results obtained in the case studies analyzed for the present work, it can be stated that ANN applications tend to focus on short-term mechanical properties, while long-term durability parameters remain insufficiently addressed. Hence, for future work, accelerated aging data (including aspects such as chloride penetration, carbonation depth, and freeze–thaw cycles) should be used in an ANN to improve predictive reliability in the mechanisms affecting the service life of these complex materials.

Furthermore, multivariate prediction frameworks capable of simultaneous eco-mechanical optimization are needed to support practical mix design. Moreover, greater attention should be paid to the regional adaptation of SCM strategies, especially in low-carbon pathways aligned with circular economy principles. Finally, a key area for development is that open-source tools and collaborative platforms could enhance model transparency, benchmarking, and widespread adoption across industry and academia. While recent studies demonstrate the promise of ANN–LCA integration, their practical translation into broader scientific and industrial applications requires further refinement. In particular, harmonized data standards, collaborative open-access datasets, and the inclusion of economic considerations remain underdeveloped. Table 5 presents a synthesis of these recommendations to guide future research directions.

## 6. Conclusions

The integration of ANNs and LCA represents a powerful methodological convergence for advancing the sustainable design of cementitious composites. This review has systematically examined recent studies that implement ANN–LCA frameworks to optimize mix design formulations containing SCMs. By analyzing diverse case studies with varying materials, modeling strategies, and environmental scopes, this study distills key patterns, performance metrics, and technical insights. The conclusions drawn below summarize the core findings and implications for the scientific and engineering community:

- ANN–LCA integration enhances multi-objective optimization. The reviewed studies demonstrate that combining ANNs with LCA enables the simultaneous optimization of mechanical performance and environmental impact. This synergy facilitates agile design of concrete mixes tailored to strength, cost, and carbon footprint criteria.
- SCMs offer significant environmental benefits when properly optimized; materials such as fly ash, slag, rice husk ash, and glass powder have shown reductions in GWP ranging from 30% to 78%, depending on replacement ratios and process conditions, without compromising compressive strength.
- FNN and MLP architectures are the most commonly used; most studies employed these simple but effective ANN structures trained on experimentally derived data. It is important to highlight that further integration with optimization algorithms (e.g., TLBO, cuckoo, and ABC) has shown enhanced predictive accuracy and a wide diversity of possible solutions.
- The cradle-to-gate LCA approach is predominant, but assumptions have a substantial influence. The dominant use of cradle-to-gate attributional LCA ensures consistency, yet system boundaries and allocation strategies significantly influence comparative assessments, particularly when dealing with industrial waste valorization. Further studies are needed to expand these approaches and incorporate the impacts of other material life-cycle phases, such as in-use application.
- The ANN–LCA framework strengthens intelligent material design and supports a circular economy. This integration not only enables the selection of mixes with lower environmental impact and solid mechanical performance but also promotes the use of industrial by-products. As a result, it contributes to the development of more sustainable construction practices aligned with global climate goals and the pursuit of carbon neutrality.
- Policymakers are strongly advised to implement measures such as mandating LCA disclosures, updating building codes for SCM use, funding AI-driven material research, providing carbon incentives, standardizing LCA methods, supporting open data, and promoting pilot projects and cross-sector collaboration to accelerate the adoption of LCA–ANN integration for optimizing the design of sustainable cementitious composites incorporating SCM.

## Figures and Tables

**Table 1:** Representative elementary flows per 1 ton of clinker.

Flow	Typical Value	Source/Description	References
Thermal energy	3200–3800 MJ	Rotary kilns; fossil/alternative fuels	[46,47]
Electricity	90–120 kWh	Grinding and homogenization processes	[48,49]
Process emissions (CaCO_3_ → CaO + CO_2_)	~520–580 kg CO_2_	Calcite content ~75% by mass	[50,51]
NO_x_ emissions	1.8–3.2 kg	Kiln temperatures ≥ 1450 °C	[52,53]
SO_2_ emissions	0.4–2.1 kg	Sulfur content in fuel and raw materials	[54,55]

**Table 2:** Relevant LCA impact categories for clinker-based cementitious materials.

Impact Category	Indicator (Unit)	Typical Contribution from Clinker (%)	References
GWP (100y)	kg CO_2_-eq	60–80%	[48,49,51]
Cumulative Energy Demand	MJ	70–85%	[46,47]
Acidification Potential	kg SO_2_-eq	40–60%	[53,54]
Eutrophication Potential	kg PO_4_^3−^-eq	20–40%	[52,55]
Photochemical Ozone Formation	kg C_2_H_4_-eq	30–50%	[50]
Particulate Matter Formation	kg PM_10_-eq	50–70%	[51,54]
Water Scarcity Footprint	m^3^ H_2_O-eq	5–20%	[47]
Abiotic Depletion (Fossil)	kg Sb-eq	70–90%	[48,49]
Land Use	Pt (points)	30–60%	[53,55]

**Table 3:** Case Study Overview: SCMs, ANN Architectures, LCA Approaches, and Key Findings.

Ref.	SCMs Employed	Composite Considered	ANN-Based Model	LCA Type	System BoundAry [Data Source]	Optimization Objective	Key Findings
[24]	Mortars with Glass Powder	Mortar	MLP	Explicit	Cradle-to-Gate [previous literature]	Hydration Mode Prediction	Glass powder reduces impacts; ANN accurately classifies hydration behavior.
[7]	Slag, Fly Ash, Palm Oil FA, Ceramic Powder	Mortar and concrete	FNN	Explicit	Cradle-to-Gate [Inventory of carbon and energy database]	Minimize CO_2_ and Energy; Maximize Strength	Optimized mixes showed lower emissions with high durability.
[22]	Glass Powder	Concrete	Backpropagation neural network, GAN	Implicit	Cradle-to-Gate [previous literature]	Balance Cost, Strength, Durability, and Impact	Glass blends achieved strong performance and lower costs.
[20]	Fly Ash, Slag, Silica Fume, Metakaolin	Concrete	CNN	Explicit	Cradle-to-Gate [previous literature]	Strength–Cost-Impact Tradeoff	Mixtures > 40 MPa; 20% less CO_2_ with <10% cost rise.
[21]	Fly Ash, Slag, Rice Husk Ash	Concrete	FNN	Explicit	Cradle-to-Gate [Ecoinvent database]	Minimize CO_2_, Energy; Maintain Strength	Up to 53% CO_2_ cut with ≥35 MPa strength.
[140]	Fly Ash, Slag, Glass Powder	Geopolymer concrete	FNN	Explicit	Cradle-to-Gate [previous literature]	Predict Strength, CO_2_, Energy	Fly Ash + slag optimal; >30 MPa, 61% CO_2_ cut.
[141]	Fly Ash, Slag, CKD	Concrete	MLP	Explicit	Cradle-to-Gate [previous literature]	Predict Strength and Environmental Impacts	CKD + slag mixes achieved 30 MPa with 45–65% GWP cut.
[28]	RHA, Fly Ash, Slag	Geopolymer concrete	MLP	Explicit	Cradle-to-Gate [EcoInvent database]	Pareto-optimal Environmental–Mechanical Mix	≥35 MPa with GWP cut up to 59%.
[27]	Fly Ash, Silica Fume	Concrete	MLP	Explicit	Cradle-to-Gate [previous literature]	High Strength + Environmental Saving	Strength > 40 MPa with 63% CO_2_ reduction.
[142]	Fly Ash (FA-I, FA-II), GGBS	Mortar	FNN	Explicit	Cradle-to-Gate [EcoInvent database]	Minimize GWP/Strength Ratio	60% GWP cut; fly ash enhances flow, strength recovery.
[143]	Fly Ash	Concrete	PINN	Implicit	Cradle-to-Gate [previous literature]	Temperature, Equivalent Age, Degree of Hydration, and Heat Generation Rate	63% CO_2_ and 73% water binding reduction.
[26]	Sugarcane Bagasse Ash (SCBA), Fly Ash	Concrete	ANN	Explicit	Cradle-to-Gate [Intergovernmental panel on climate change guidelines]	SHAP Interpretation	Up to 40 MPa, strong emission reduction.
[25]	Fly Ash In Geopolymer concrete (FA-GPC)	Geopolymer concrete	ANN	Explicit	Cradle-to-Gate [previous literature]	Strength and Emission Prediction	78% CO_2_ reduction, 90 MPa strength.
[144]	Fly Ash, Slag, Silica Fume	Concrete	MLP	Explicit	Cradle-to-Gate [National statistical data from Australia]	Impact Prediction from Mix Design	RA + SCM synergy, allocation scenario insights.

**Table 4:** Performance of SCM-optimized cementitious composites.

Ref.	Strength retention	Environmental savings	ANN model performance
[24]	Cement composites with 50% glass powder exhibited a 28 to 57% reduction in compressive strength at 90 days compared to conventional mortars; however, the strength continued to increase over time due to pozzolanic reactions.	Substitution with glass powder resulted in approximately 50% lower CO_2_ emissions for Portland cement blends and up to 80% reduction for slag-based blends, highlighting significant environmental benefits.	The model achieved very high predictive accuracy (average R^2^ ≈ 0.96), reliably identifying hydration modes and closely matching the experimental heat of hydration data.
[7]	Cement composites with 30 to 50% fly ash or slag replacement maintained over 80/90% of their 28-day compressive strength at later ages, compared to plain cement mixes that showed faster strength decline in aggressive environments.	High-volume fly ash mixes reduced embodied carbon from 436.8 kg CO_2_/m^3^ (ordinary cement mortar) to 45.5 kg CO_2_/m^3^ and reduced embodied energy from 2793 MJ/m^3^ to 881.2 MJ/m^3^, giving nearly 90% lower emissions and 68% lower energy demand.	The neural network predicted embodied carbon and energy with R^2^ values above 0.97, showing high accuracy in optimizing mixture design for both mechanical and environmental performance.
[22]	When glass powder replacement increases from 10% to 50%, compressive strength falls to only 1.64%, and optimized mixtures in the Pareto set commonly lie in the 30 to 60 MPa range (many top solutions 42 to 56 MPa).	A 32% decrease in the overall environmental indicator and a 18% drop in life-cycle material cost when glass powder replacement goes from 10% to 50%.	After swarm-based hyperparameter tuning, the ANN predicted compressive strength with R^2^ values higher than 0.94.
[20]	The SCM-optimized mixtures in the study retain about 45 MPa at 28 days and 55 MPa at 56 days, outperforming the comparison methods.	Integrating SCMs and the optimization routine resulted in a roughly 20% reduction in CO_2_ emissions, with reported optimized mixes ranging from 270 to 280 kg CO_2_/m^3^ of concrete.	The hybrid ensemble achieved an R^2^ score of approximately 0.86 for predicting compressive strength.
[21]	With zeolite replacing ~25–30% of cement, unconfined compressive strength reached 2.5 to 2.8 MPa, and the optimized replacement window of 30–45% maintained high strength.	Relative to cement-only mixes, zeolite-blended mixes reduce the total weighted life-cycle impact by around 35%, including 34% lower human-health damage and 49% lower ecosystem damage.	The best back-propagation model achieved an R^2^ ≈ of 0.999 across 10 runs, accurately predicting strength values.
[140]	SCM-optimized cement composites retained over 90% of the compressive strength compared to control mixes, even with up to 30% clinker replacement.	SCM inclusion reduced CO_2_ emissions by 25 to 40% and energy consumption by around 20% relative to ordinary Portland cement composites.	R^2^ values greater than 0.95, demonstrating high predictive accuracy in strength and environmental performance estimation.
[141]	SCM-optimized cement composites maintained compressive strength within 90 to 100% of control mixes while reducing clinker content.	Incorporation of SCMs reduced embodied CO_2_ emissions by approximately 20 to 35% compared to ordinary Portland cement mixes.	The ANN achieved high predictive accuracy with R^2^ values above 0.95 and mean squared errors below 0.01 in forecasting strength and environmental indicators.
[28]	SCM-optimized concrete with rice husk ash achieved compressive strengths up to 104 MPa.	The optimal SCM mix reduced the carbon footprint to 289.85 kg CO_2_ eq, which is approximately 33% lower than that of ordinary Portland cement concrete (386 kg CO_2_ eq), while also lowering the acidification potential to 0.66 kg SO_2_ eq and water consumption to 5.77 m^3^ per cubic meter of concrete.	The proposed ANN model achieved R^2^ scores higher than 0.96, outperforming other AI techniques and earlier models, demonstrating excellent predictive capability for compressive strength.
[27]	The SCM-optimized concrete (30% fly ash + 15% alccofine) achieved the highest compressive strength, exceeding ordinary Portland cement concrete by more than 20% at 28 days.	Optimized mixes reduced the GWP and other impact categories by over 30% compared to ordinary Portland cement concrete.	The ANN achieved R^2^ values above 0.90 across training, validation, and testing sets, confirming high accuracy in predicting compressive strength.
[142]	SCM-optimized cement composites with 50 to 70% cement replacement achieved up to 24% higher 28-day and 13% higher 90-day compressive strength compared to binary blends, with the best performance when fly ash content was limited to 20–30%.	Replacing 70% of cement with fly ash and slag reduced CO_2_ emissions by 380 kg/m^3^ (63%), giving the lowest global warming potential per unit strength in the ternary mixes with 10–20% fly ash.	The ANN achieved a mean square error of 0.0997 and an R^2^ value between 0.96 and 0.99, showing high accuracy in predicting eco-mechanical performance.
[143]	SCM-optimized cement composites retained up to 95% of compressive strength at 28 days compared to control mixes while reducing clinker content.	These mixes achieved up to 40% reduction in GWP and 35% lower embodied energy relative to conventional cement.	The proposed ANN model exhibited predictive errors of nearly zero for both compressive strength and environmental indicators.
[26]	SCM-optimized cement composites retained over 90% of reference compressive strength at 28 days while incorporating high levels of SCMs.	LCA showed 25 to 40% reductions in CO_2_ emissions and energy demand compared to conventional mixes.	The ANN achieved a prediction accuracy above 95% (R^2^ > 0.95) for compressive strength across different SCM combinations.
[25]	SCM-optimized cement composites retained over 90% of the compressive strength at 28 days while reducing clinker content.	The optimized mixes achieved up to 35% reduction in CO_2_ emissions and significant decreases in energy demand compared to conventional cement.	The ANN model demonstrated high predictive accuracy with an R^2^ above 0.95 for both strength and environmental indicators.
[144]	SCM-optimized cement composites retained up to 90–100% of compressive strength compared to control mixes even at high clinker replacement levels.	SCM-optimized cement composites achieved up to 40% reduction in CO_2_ emissions and energy demand relative to conventional cement.	The ANN model demonstrated R^2^ values above 0.95 with low prediction errors, confirming high accuracy in predicting.

**Table 5:** Key recommendations for advancing ANN–LCA research in cementitious systems.

Recommendation Area	Actions	Impact
Data standardization in LCA–ANN studies	Establish common input/output protocols (e.g., SCM proportions, curing conditions, compressive strength, and CO_2_ emissions) and reporting guidelines (aligned with ISO 14040/14044).	Improves comparability and reproducibility across studies; reduces methodological preferences.
Development of open-access SCM datasets	Create shared repositories integrating experimental results, material characterizations, and regional energy mixes, and encourage FAIR principles (Findable, Accessible, Interoperable, Reusable).	Enables robust ANN training, reduces redundancy, and fosters collaboration between academia and industry.
Integration of economic and financial-related assessments	Extend ANN–LCA frameworks to include cost parameters (e.g., embodied energy cost, maintenance, and service life), and combine environmental and economic metrics in multi-objective optimization.	Supports decision-making that balances sustainability with financial feasibility, increasing industry uptake.

## Data Availability

No new data were created or analyzed in this study. Data sharing is not applicable to this article.

## Abbreviations

The following abbreviations are used in this manuscript:

**AI**	**Artificial Intelligence**
ANNs	Artificial Neural Networks
CO	Carbon Monoxide
CO_2_	Carbon Dioxide
CNNs	Convolutional Neural Networks
DDNNs	Data-Driven Neural Networks
DL	Deep Learning
DNNs	Deep Neural Networks
ELMs	Extreme Learning Machines
FNNs	Feedforward Neural Networks
GANs	Generative Adversarial Networks
GNNs	Graph Neural Networks
GWP	Global Warming Potential
LCA	Life-Cycle Assessment
LCI	Life-Cycle Inventory
LCIA	Life-Cycle Impact Assessment
ML	Machine Learning
MLPs	Multilayer Perceptrons
NO_x_	Nitrogen Oxides
OPC	Ordinary Portland Cement
PINNs	Physics-Informed Neural Networks
PRISMA	Preferred Reporting Items for Systematic Reviews and Meta-Analyses
RBFNs	Radial Basis Function Networks
ResNets	Residual Neural Networks
RNNs	Recurrent Neural Networks
SCMs	Supplementary Cementitious Materials
SNNs	Shallow Neural Networks
SO_2_	Sulfur Dioxide
TANNs	Thermodynamics-Based Artificial Neural Networks
TNNs	Transformer Neural Networks

## References

[1] Kumar D., Alam M., Sanjayan J., Duan W., Zhang L., Shah S.P. (2023). A Novel Concrete Mix Design Methodology. Nanotechnology in Construction for Circular Economy.

[2] Pineda A., Peñabaena-Niebles R., Martínez-Arguelles G., Polo-Mendoza R. (2025). Development of OptiCon: A mathematical model with a graphical user interface for designing sustainable Portland cement concrete mixes with budget constraint. Inventions.

[3] Tee K.F., Mostofizadeh S. (2021). A mini review on properties of Portland cement concrete with geopolymer materials as partial or entire replacement. Infrastructures.

[4] Walubita L.F., Martinez-Arguelles G., Polo-Mendoza R., Ick-Lee S., Fuentes L. (2022). Comparative environmental assessment of rigid, flexible, and perpetual pavements: A case study of Texas. Sustainability.

[5] Guerrero-Bustamante O., Guillen A., Moreno-Navarro F., Rubio-Gámez M.C., Sol-Sánchez M. (2025). Cold mix asphalt for sustainable bituminous sub-ballast for railway: Mechanical, vibratory and environmental assessment. J. Clean. Prod..

[6] Habert G., Miller S.A., John V.M., Provis J.L., Favier A., Horvath A., Scrivener K.L. (2020). Environmental impacts and decarbonization strategies in the cement and concrete industries. Nat. Rev. Earth Environ..

[7] Faridmehr I., Nehdi M.L., Nikoo M., Huseien G.F., Ozbakkaloglu T. (2021). Life-cycle assessment of alkali-activated materials incorporating industrial byproducts. Materials.

[8] Gupta S., Chaudhary S. (2022). State of the art review on supplementary cementitious materials in India—II: Characteristics of SCMs, effect on concrete and environmental impact. J. Clean. Prod..

[9] Sivakrishna A., Adesina A., Awoyera P.O., Rajesh Kumar K. (2020). Green concrete: A review of recent developments. Mater. Today Proc..

[10] Al-Hamrani A., Kucukvar M., Alnahhal W., Mahdi E., Onat N.C. (2021). Green concrete for a circular economy: A review on sustainability, durability, and structural properties. Materials.

[11] Camargo-Pérez N.R., Abellán-García J., Fuentes L. (2023). Use of rice husk ash as a supplementary cementitious material in concrete mix for road pavements. J. Mater. Res. Technol..

[12] Mosquera C.H., Acosta M.P., Rodríguez W.A., Mejía-España D.A., Torres J.R., Martinez D.M., Abellán-García J. (2025). ANN-based analysis of the effect of SCM on recycled aggregate concrete. Struct. Concr..

[13] Ahmed A. (2024). Assessing the effects of supplementary cementitious materials on concrete properties: A review. Discov. Civ. Eng..

[14] Shanahan N., Tran V., Williams A., Zayed A. (2016). Effect of SCM combinations on paste rheology and its relationship to particle characteristics of the mixture. Constr. Build. Mater..

[15] DeRousseau M.A., Kasprzyk J.R., Srubar W.V. (2018). Computational design optimization of concrete mixtures: A review. Cem. Concr. Res..

[16] Samad S., Shah A. (2017). Role of binary cement including supplementary cementitious material (SCM), in production of environmentally sustainable concrete: A critical review. Int. J. Sustain. Built Environ..

[17] Papadakis V.G., Tsimas S. (2002). Supplementary cementing materials in concrete. Cem. Concr. Res..

[18] Pang L., Liu Z., Wang D., An M. (2022). Review on the application of supplementary cementitious materials in self-compacting concrete. Crystals.

[19] Guerrero-Bustamante O., Camargo R., Duque J., Martinez-Arguelles G., Polo-Mendoza R., Acosta C., Murillo M. (2025). Designing sustainable asphalt pavement structures with a cement-treated base (CTB) and recycled concrete aggregate (RCA): A case study from a developing country. Designs.

[20] Mungle N.P., Mate D.M., Mankar S.H., Tale V.T., Vairagade V.S., Shelare S.D. (2024). Applications of computational intelligence for predictive modeling of properties of blended cement sustainable concrete incorporating various industrial byproducts towards sustainable construction. Asian J. Civ. Eng..

[21] Nasrollahpour S., Tanhadoust A., Kaur Brar S., MolaAbasi H., Nehdi M.L., Ataee O. (2024). Multi-objective optimization of sustainable cement-zeolite improved sand based on life cycle assessment and artificial intelligence. F1000Research.

[22] Miao X., Zhao H., Peng L., Zhao Y. (2025). Eco-friendly intelligent mixture design of glass powder concrete: A life cycle perspective with hybrid machine learning and generative adversarial networks. J. Build. Eng..

[23] Datta S.D., Sarkar M.M., Rakhe A.S., Aditto F.S., Sobuz M.H.R., Shaurdho N.M.N., Nijum N.J., Das S. (2024). Analysis of the characteristics and environmental benefits of rice husk ash as a supplementary cementitious material through experimental and machine learning approaches. Innov. Infrastruct. Solut..

[24] Boukhelf F., Targino D.L.L., Benzaama M.H., Babadopulos L.F.d.A., El Mendili Y. (2023). Insight into the behavior of mortars containing glass powder: An artificial neural network analysis approach to classify the hydration modes. Materials.

[25] Siddiq M.U., Anwar M.K., Almansour F.H., Qurashi M.A., Adeel M. (2025). AI-driven optimization of fly ash-based geopolymer concrete for sustainable high strength and co2 reduction: An application of hybrid taguchi–grey–ann approach. Buildings.

[26] Rizwan V., Ibrahim S.M., Zaheer M.M., Rehman A.ur. (2025). Explainable artificial intelligence-based compressive strength optimization and life-cycle assessment of eco-friendly sugarcane bagasse ash concrete. Environ. Sci. Pollut. Res..

[27] Padavala S.S.A.B., Noolu V., Paluri Y., Bijivemula S.K.R., Akula U.K. (2024). A study on the synthesis and performance evaluation of fly ash and alccofine as sustainable cementitious materials. Sci. Rep..

[28] Onyelowe K.C., Ebid A.M., Mahdi H.A., Riofrio A., Eidgahee D.R., Baykara H., Soleymani A., Kontoni D.-P.N., Shakeri J., Jahangir H. (2022). Optimal compressive strength of RHA ultra-high-performance lightweight concrete (UHPLC) and its environmental performance using life cycle assessment. Civ. Eng. J..

[29] Juenger M.C.G., Siddique R. (2015). Recent advances in understanding the role of supplementary cementitious materials in concrete. Cem. Concr. Res..

[30] Golewski G.L. (2019). The influence of microcrack width on the mechanical parameters in concrete with the addition of fly ash: Consideration of technological and ecological benefits. Constr. Build. Mater..

[31] Abdalla L.B., Ghafor K., Mohammed A. (2019). Testing and Modeling the young age compressive strength for high workability concrete modified with PCE polymers. Results Mater..

[32] Golewski G.L. (2018). Effect of curing time on the fracture toughness of fly ash concrete composites. Compos. Struct..

[33] Aprianti S.E. (2017). A huge number of artificial waste material can be supplementary cementitious material (SCM) for concrete production—A review part II. J. Clean. Prod..

[34] Wang T., Lee I.-S., Kendall A., Harvey J., Lee E.-B., Kim C. (2012). Life cycle energy consumption and GHG emission from pavement rehabilitation with different rolling resistance. J. Clean. Prod..

[35] Mattinzioli T., Lo Presti D., Jiménez del Barco Carrión A. (2022). A Critical review of life cycle assessment benchmarking methodologies for construction materials. Sustain. Mater. Technol..

[36] Miller S.A., Cunningham P.R., Harvey J.T. (2019). Rice-based ash in concrete: A review of past work and potential environmental sustainability. Resour. Conserv. Recycl..

[37] (2006). Environmental Management-Life Cycle Assessment-Principles and Framework.

[38] (2006). Environmental Management—Life Cycle Assessment—Requirements and Guidelines.

[39] Cheung W.M., Marsh R., Griffin P.W., Newnes L.B., Mileham A.R., Lanham J.D. (2015). Towards cleaner production: A roadmap for predicting product end-of-life costs at early design concept. J. Clean. Prod..

[40] Kokare S., Oliveira J.P., Godina R. (2023). Life cycle assessment of additive manufacturing processes: A review. J. Manuf. Syst..

[41] Polo-Mendoza R., Martinez-Arguelles G., Peñabaena-Niebles R., Covilla-Valera E. (2024). Neural networks implementation for the environmental optimisation of the recycled concrete aggregate inclusion in warm mix asphalt. Road Mater. Pavement Des..

[42] Mohammadi A., Ramezanianpour A.M. (2023). Investigating the environmental and economic impacts of using supplementary cementitious materials (SCMs) using the life cycle approach. J. Build. Eng..

[43] Chen C., Habert G., Bouzidi Y., Jullien A., Ventura A. (2010). LCA Allocation procedure used as an incitative method for waste recycling: An application to mineral additions in concrete. Resour. Conserv. Recycl..

[44] Shin K., Kim J., Hwang Y.W., Kim B.C., Kim D.H. (2024). LCA-based environmental benefit allocation between steel and cement industries in steel byproduct recycling. Environ. Eng. Res..

[45] Bhagath Singh G.V.P., Durga Prasad V. (2024). Environmental Impact of concrete containing high volume fly ash and ground granulated blast furnace slag. J. Clean. Prod..

[46] Çankaya S., Pekey B. (2020). Application of Scenario analysis for assessing the environmental impacts of thermal energy substitution and electrical energy efficiency in clinker production by life cycle approach. J. Clean. Prod..

[47] Galvez-Martos J.-L., Schoenberger H. (2014). An analysis of the use of life cycle assessment for waste co-incineration in cement kilns. Resour. Conserv. Recycl..

[48] Cherni M., Sebei A., Amor B., Hssine N., Hajjaji N. (2024). Critical reviews and benchmarking tunisian clinker and cement with life cycle assessment results. Case Stud. Constr. Mater..

[49] Salaripoor H., Yousefi H., Abdoos M. (2025). Life cycle environmental assessment of refuse-derived fuel (RDF) as an alternative to fossil fuels in cement production: A sustainable approach for mitigating carbon emissions. Fuel Commun..

[50] Shirkhani A., Kouchaki-Penchah H., Azmoodeh-Mishamandani A. (2018). Environmental and exergetic impacts of cement production: A case study. Environ. Prog. Sustain. Energy.

[51] Wolde M.G., Khatiwada D., Bekele G., Palm B. (2024). A life cycle assessment of clinker and cement production in Ethiopia. Clean. Environ. Syst..

[52] Seyler C., Hellweg S., Monteil M., Hungerbühler K. (2005). Life cycle inventory for use of waste solvent as fuel substitute in the cement industry—A multi-input allocation model (11 Pp). Int. J. Life. Cycle. Assess..

[53] Mishra U.C., Sarsaiya S., Gupta A. (2022). A Systematic review on the impact of cement industries on the natural environment. Environ. Sci. Pollut. Res..

[54] Lei Y., Zhang Q., Nielsen C., He K. (2011). An inventory of primary air pollutants and CO2 Emissions from cement production in China, 1990–2020. Atmos. Environ..

[55] Sudhakar C.V., Reddy G.U. (2023). Impacts of cement industry air pollutants on the environment and satellite data applications for air quality monitoring and management. Environ. Monit. Assess..

[56] Tam V.W.Y., Soomro M., Evangelista A.C.J. (2018). A review of recycled aggregate in concrete applications (2000–2017). Constr. Build. Mater..

[57] Henkensiefken R., Bentz D., Nantung T., Weiss J. (2009). Volume change and cracking in internally cured mixtures made with saturated lightweight aggregate under sealed and unsealed conditions. Cem. Concr. Compos..

[58] Guerrero-Bustamante O., Guillen A., Moreno-Navarro F., Rubio-Gámez M.C., Sol-Sánchez M. (2025). Suitable granular road base from reclaimed asphalt pavement. Materials.

[59] Abioye S.O., Oyedele L.O., Akanbi L., Ajayi A., Davila Delgado J.M., Bilal M., Akinade O.O., Ahmed A. (2021). Artificial intelligence in the construction industry: A review of present status, opportunities and future challenges. J. Build. Eng..

[60] Sarker I.H. (2022). AI-based modeling: Techniques, applications and research issues towards automation, intelligent and smart systems. SN Comput. Sci..

[61] Xu Y., Liu X., Cao X., Huang C., Liu E., Qian S., Liu X., Wu Y., Dong F., Qiu C.-W. (2021). Artificial intelligence: A powerful paradigm for scientific research. Innovation.

[62] Emmert-Streib F., Yli-Harja O., Dehmer M. (2020). Artificial intelligence: A clarification of misconceptions, myths and desired status. Front. Artif. Intell..

[63] Mukhamediev R.I., Popova Y., Kuchin Y., Zaitseva E., Kalimoldayev A., Symagulov A., Levashenko V., Abdoldina F., Gopejenko V., Yakunin K. (2022). Review of artificial intelligence and machine learning technologies: Classification, restrictions, opportunities and challenges. Mathematics.

[64] Sarker I.H. (2021). Machine learning: Algorithms, real-world applications and research directions. SN Comput. Sci..

[65] Pugliese R., Regondi S., Marini R. (2021). Machine learning-based approach: Global Trends, research directions, and regulatory standpoints. Data Sci. Manag..

[66] Janiesch C., Zschech P., Heinrich K. (2021). Machine learning and deep learning. Electron. Mark..

[67] Sun Y., Vong C.M., Wang S. (2025). Adversarial de-overlapping learning machines for supervised and semi-supervised learning. Int. J. Mach. Learn. Cybern..

[68] Takahashi K., Takahashi L. (2024). Supervised machine learning. Materials Informatics and Catalysts Informatics.

[69] Soobramoney J., Chifurira R., Zewotir T., Chinhamu K. (2025). Identifying the intents behind website visits by employing unsupervised machine learning models. Ann. Data Sci..

[70] Pascoal F., Branco P., Torgo L., Costa R., Magalhães C. (2025). Definition of the microbial rare biosphere through unsupervised machine learning. Commun. Biol..

[71] Jiang H., Wang G., Li S., Zhang J., Yan L., Xu X. (2025). Hierarchical reinforcement learning based on macro actions. Complex Intell. Syst..

[72] Chan J.H., Liu K., Chen Y., Sagar A.S.M.S., Kim Y.-G. (2024). Reinforcement learning-based drone simulators: Survey, practice, and challenge. Artif. Intell. Rev..

[73] Yaghoubi E., Yaghoubi E., Khamees A., Vakili A.H. (2024). A systematic review and meta-analysis of artificial neural network, machine learning, deep learning, and ensemble learning approaches in field of geotechnical engineering. Neural Comput. Appl..

[74] Muñoz-Zavala A.E., Macías-Díaz J.E., Alba-Cuéllar D., Guerrero-Díaz-de-León J.A. (2024). A literature review on some trends in artificial neural networks for modeling and simulation with time series. Algorithms.

[75] Alzubaidi L., Zhang J., Humaidi A.J., Al-Dujaili A., Duan Y., Al-Shamma O., Santamaría J., Fadhel M.A., Al-Amidie M., Farhan L. (2021). Review of deep learning: Concepts, CNN architectures, challenges, applications, future directions. J. Big Data.

[76] Sarker I.H. (2021). Deep learning: A comprehensive overview on techniques, taxonomy, applications and research directions. SN Comput. Sci..

[77] Alvarez-Rodríguez S., Peña-Lecona F.G. (2023). Artificial neural networks with machine learning design for a polyphasic encoder. Sensors.

[78] Kufel J., Bargieł-Łączek K., Kocot S., Koźlik M., Bartnikowska W., Janik M., Czogalik Ł., Dudek P., Magiera M., Lis A. (2023). What is machine learning, artificial neural networks and deep learning?—Examples of practical applications in medicine. Diagnostics.

[79] Polo-Mendoza R., Martinez-Arguelles G., Peñabaena-Niebles R., Duque J. (2024). Development of a machine learning (ML)-based computational model to estimate the engineering properties of Portland cement concrete (PCC). Arab. J. Sci. Eng..

[80] Tóth B., Guerrero-Bustamante O., Murillo M., Duque J., Polo-Mendoza R. (2025). Development of Mathematical and computational models for predicting agricultural soil–water management properties (ASWMPs) to optimize intelligent irrigation systems and enhance crop resilience. Agronomy.

[81] Wang G., Fan F.-L. (2025). Dimensionality and dynamics for next-generation artificial neural networks. Patterns.

[82] Liu L., Xue J., Meng Y., Xu T., Cong M., Ding Y., Yang Y. (2025). Application of artificial neural networks to acoustic composites: A review. Mater. Today Commun..

[83] Sharma N., Joshi S., Goswami P. (2025). An Enhanced artificial neural network approach for solving nonlinear fractional-order differential equations. Partial. Differ. Equ. Appl. Math..

[84] Almonacid F., Fernandez E.F., Mellit A., Kalogirou S. (2017). Review of Techniques based on artificial neural networks for the electrical characterization of concentrator photovoltaic technology. Renew. Sustain. Energy Rev..

[85] Sevakula R.K., Au-Yeung W.M., Singh J.P., Heist E.K., Isselbacher E.M., Armoundas A.A. (2020). State-of-the-art machine learning techniques aiming to improve patient outcomes pertaining to the cardiovascular system. J. Am. Heart. Assoc..

[86] Malekjani N., Kharaghani A., Tsotsas E. (2025). Physics-informed and data-driven neural networks with dimensional and non-dimensional inputs for single-droplet evaporation: Investigating the role of increasing physical complexity in predictive ability. Chem. Eng. Sci..

[87] Xu R.-Z., Cao J.-S., Luo J.-Y., Ni B.-J., Fang F., Liu W., Wang P. (2024). Data-driven neural networks for biological wastewater resource recovery: Development and challenges. J. Clean. Prod..

[88] Nguyen H.-T., Cheah C.C. Data-driven neural network-based learning for regression problems in robotics. Proceedings of the IECON 2020 The 46th Annual Conference of the IEEE Industrial Electronics Society.

[89] Ouyang W., Li G., Chen L., Liu S.-W. (2024). Machine learning-based soil–structure interaction analysis of laterally loaded piles through physics-informed neural networks. Acta Geotech..

[90] Katsikis D., Muradova A.D., Stavroulakis G.E. (2022). A gentle introduction to physics-informed neural networks, with applications in static rod and beam problems. J. Adv. Appl. Comput. Math..

[91] Polo-Mendoza R., Mašín D., Duque J. (2025). Integrating the grading entropy theory (GET) into a physics-informed neural network (PINN) to predict soil hydraulic properties. Results Eng..

[92] Madhiarasan M., Louzazni M. (2022). Analysis of artificial neural network: Architecture, types, and forecasting applications. J. Electr. Comput. Eng..

[93] Montesinos López O.A., Montesinos López A., Crossa J. (2022). Fundamentals of artificial neural networks and deep learning. Multivariate Statistical Machine Learning Methods for Genomic Prediction.

[94] Tran D., Tham A.W. (2025). Accuracy comparison between feedforward neural network, support vector machine and boosting ensembles for financial risk evaluation. J. Risk Financ. Manag..

[95] Tiwari S., Ahn H., Reddy M.H., Park N., Reddy N.G.S. (2025). Mechanical property prediction of industrial low-carbon hot-rolled steels using artificial neural networks. Materials.

[96] Bartczak N., Glanowska M., Kowalewicz K., Kunin M., Susik R. (2025). Fall detection based on recurrent neural networks and accelerometer data from smartphones. Appl. Sci..

[97] Belloni E., Forconi F., Lozito G.M., Palermo M., Quercio M., Riganti Fulginei F. (2025). Development of recurrent neural networks for thermal/electrical analysis of non-residential buildings based on energy consumptions data. Energies.

[98] Sawicki P., Dybała B. (2025). Enhancing powder bed fusion—Laser beam process monitoring: Transfer and classic learning techniques for convolutional neural networks. Materials.

[99] Sreejith R., Ramasamy R.K., Mohd-Isa W.-N., Abdullah J. Enhanced lung disease detection using double denoising and 1D convolutional neural networks on respiratory sound analysis. Proceedings of the International Conference on Sustainable Computing and Green Technologies (SCGT’2025).

[100] Gao M., Qi D., Mu H., Chen J. (2021). A transfer residual neural network based on ResNet-34 for detection of wood knot defects. Forests.

[101] Thorpe M., van Gennip Y. (2023). Deep limits of residual neural networks. Res. Math. Sci..

[102] Abed M., Imteaz M.A., Ahmed A.N., Huang Y.F. (2023). A novel application of transformer neural network (TNN) for estimating pan evaporation rate. Appl. Water Sci..

[103] Mamatov N.S., Niyozmatova N.A., Abdullaev S.S., Samijonov A.N., Erejepov K.K. Speech recognition based on transformer neural networks. Proceedings of the 2021 International Conference on Information Science and Communications Technologies (ICISCT).

[104] Khemani B., Patil S., Kotecha K., Tanwar S. (2024). A review of graph neural networks: Concepts, architectures, techniques, challenges, datasets, applications, and future directions. J. Big Data.

[105] Zhou J., Cui G., Hu S., Zhang Z., Yang C., Liu Z., Wang L., Li C., Sun M. (2020). Graph neural networks: A review of methods and applications. AI Open.

[106] Aggarwal A., Mittal M., Battineni G. (2021). Generative adversarial network: An overview of theory and applications. Int. J. Inf. Manag. Data Insights.

[107] Feng J., Feng X., Chen J., Cao X., Zhang X., Jiao L., Yu T. (2020). Generative adversarial networks based on collaborative learning and attention mechanism for hyperspectral image classification. Remote Sens..

[108] Goodfellow I., Pouget-Abadie J., Mirza M., Xu B., Warde-Farley D., Ozair S., Courville A., Bengio Y. (2020). Generative adversarial networks. Commun. ACM.

[109] Kim S., Choi J.-H., Kim N.H. (2022). Data-driven prognostics with low-fidelity physical information for digital twin: Physics-informed neural network. Struct. Multidiscip. Optim..

[110] Yuan L., Ni Y.-Q., Deng X.-Y., Hao S. (2022). A-PINN: Auxiliary physics informed neural networks for forward and inverse problems of nonlinear integro-differential equations. J. Comput. Phys..

[111] Davison A.P., Appukuttan S. (2022). A faster way to model neuronal circuitry. Elife.

[112] Fayazi M., Colter Z., Afshari E., Dreslinski R. (2021). Applications of artificial intelligence on the modeling and optimization for analog and mixed-signal circuits: A review. IEEE Trans. Circuits Syst. I Regul. Pap..

[113] Masi F., Stefanou I., Vannucci P., Maffi-Berthier V. (2021). Thermodynamics-based artificial neural networks for constitutive modeling. J. Mech. Phys. Solids.

[114] Masi F., Stefanou I. (2022). Multiscale Modeling of inelastic materials with thermodynamics-based artificial neural networks (TANN). Comput. Methods Appl. Mech. Eng..

[115] Ojha V.K., Abraham A., Snášel V. (2017). Metaheuristic design of feedforward neural networks: A review of two decades of research. Eng. Appl. Artif. Intell..

[116] Murat H.S. (2006). A Brief review of feed-forward neural networks. Commun. Fac. Sci. Univ. Ank. Ser. A2-A3 Phys. Sci. Eng..

[117] Citton O., Richert F., Biehl M. (2025). Phase transition analysis for shallow neural networks with arbitrary activation functions. Phys. A Stat. Mech. Its Appl..

[118] Fornasier M., Klock T., Mondelli M., Rauchensteiner M. (2025). Efficient identification of wide shallow neural networks with biases. Appl. Comput. Harmon. Anal..

[119] Polo-Mendoza R., Martinez-Arguelles G., Peñabaena-Niebles R. (2023). Environmental optimization of warm mix asphalt (WMA) design with recycled concrete aggregates (RCA) inclusion through artificial intelligence (AI) techniques. Results Eng..

[120] Riverol C., Pilipovik V. (2014). Assessing the failure frequency of potential hazardous incidents using radial basis function networks (RBFN). A milk pasteurization unit as study case. Food Control.

[121] Riverol-Cañizares C., Pilipovik V. (2010). The use of radial basis function networks (RBFN) to predict critical water parameters in desalination plants. Expert Syst. Appl..

[122] Shomope I., Tawalbeh M., Al-Othman A., Almomani F. (2025). Predicting biohydrogen production from dark fermentation of organic waste biomass using multilayer perceptron artificial neural network (MLP–ANN). Comput. Chem. Eng..

[123] Sumayli A. (2023). Development of Advanced machine learning models for optimization of methyl ester biofuel production from papaya oil: Gaussian process regression (GPR), multilayer perceptron (MLP), and K-nearest neighbor (KNN) regression models. Arab. J. Chem..

[124] Abu Al-Haija Q., Altamimi S., AlWadi M. (2024). Analysis of extreme learning machines (ELMs) for intelligent intrusion detection systems: A survey. Expert Syst. Appl..

[125] Rahman M., Rashid F., Roy S.K., Habib M.A. (2024). Application of extreme learning machine (ELM) forecasting model on CO2 emission dataset of a natural gas-fired power plant in Dhaka, Bangladesh. Data Brief.

[126] Franczyk B., Ludwig A., Nunez M., Treur J., Vossen G., Kozierkiewicz A. (2024). Advances in Computational Collective Intelligence.

[127] Jordan M.I., Mitchell T.M. (2015). Machine learning: Trends, perspectives, and prospects. Science.

[128] Kaplan A., Haenlein M. (2019). Siri, Siri, in my hand: Who’s the fairest in the land? On the Interpretations, illustrations, and implications of artificial intelligence. Bus. Horiz..

[129] LeCun Y., Bengio Y., Hinton G. (2015). Deep learning. Nature.

[130] Floridi L., Cowls J. (2019). A unified framework of five principles for ai in society. Harv. Data Sci. Rev..

[131] Törnberg P., Söderström O., Barella J., Greyling S., Oldfield S. (2025). Artificial intelligence and the state: Seeing like an artificial neural network. Big Data Soc..

[132] Hasson U., Nastase S.A., Goldstein A. (2020). Direct fit to nature: An evolutionary perspective on biological and artificial neural networks. Neuron.

[133] Soltoggio A., Stanley K.O., Risi S. (2018). Born to learn: The inspiration, progress, and future of evolved plastic artificial neural networks. Neural Netw..

[134] Maier H.R., Galelli S., Razavi S., Castelletti A., Rizzoli A., Athanasiadis I.N., Sànchez-Marrè M., Acutis M., Wu W., Humphrey G.B. (2023). Exploding the myths: An introduction to artificial neural networks for prediction and forecasting. Environ. Model. Softw..

[135] Joshi A., Sasumana J., Ray N.M., Kaushik V. (2021). Neural network analysis. Advances in Bioinformatics.

[136] Han D., Kwon S., Kim J., Jin W., Son H. Comprehensive Analysis for production prediction of hydraulic fractured shale reservoirs using proxy model based on deep neural network. Proceedings of the SPE Annual Technical Conference and Exhibition.

[137] Wang J., Lu S., Wang S.-H., Zhang Y.-D. (2022). A review on extreme learning machine. Multimed. Tools Appl..

[138] Moher D., Liberati A., Tetzlaff J., Altman D.G. (2010). Preferred reporting items for systematic reviews and meta-analyses: The PRISMA statement. Int. J. Surg..

[139] Page M.J., McKenzie J.E., Bossuyt P.M., Boutron I., Hoffmann T.C., Mulrow C.D., Shamseer L., Tetzlaff J.M., Akl E.A., Brennan S.E. (2021). The PRISMA 2020 statement: An updated guideline for reporting systematic reviews. BMJ.

[140] Onyelowe K.C., Kontoni D.-P.N., Ebid A.M., Dabbaghi F., Soleymani A., Jahangir H., Nehdi M.L. (2022). Multi-objective optimization of sustainable concrete containing fly ash based on environmental and mechanical considerations. Buildings.

[141] Onyelowe K.C., Ebid A.M., Mahdi H.A., Soleymani A., Jahangir H., Dabbaghi F. (2022). Optimization of green concrete containing fly ash and rice husk ash based on hydro-mechanical properties and life cycle assessment considerations. Civ. Eng. J..

[142] Radwan M.K.H., Onn C.C., Mo K.H., Yap S.P., Chin R.J., Lai S.H. (2022). Sustainable ternary cement blends with high-volume ground granulated blast furnace slag–fly ash. Environ. Dev. Sustain..

[143] Rahman M.A., Lu Y. (2024). EcoBlendNet: A physics-informed neural network for optimizing supplementary material replacement to reduce the carbon footprint during cement hydration. J. Clean. Prod..

[144] Xing W., Tam V.W., Le K.N., Hao J.L., Wang J. (2023). Life Cycle assessment of sustainable concrete with recycled aggregate and supplementary cementitious materials. Resour. Conserv. Recycl..

[145] de Paula Salgado I., Conrad F., Signorini C., Günther E., Ihlenfeldt S., Mechtcherine V. (2025). Integrating life cycle assessment (LCA) and machine learning for sustainable designs: A case study on protective layers made of mineral-bonded fiber-reinforced composites. Int. J. Life. Cycle Assess..

[146] Ateş K.T., Şahin C., Kuvvetli Y., Küren B.A., Uysal A. (2021). Sustainable Production in cement via artificial intelligence based decision support system: Case study. Case Stud. Constr. Mater..

[147] Hocine A., Kellouche Y., Ghrici M., Boukhatem B. (2018). Compressive strength prediction of limestone filler concrete using artificial neural networks. Adv. Comput. Des..

[148] Arvizu-Montes A., Alcivar-Bastidas S., Martínez-Echevarría M.J. (2025). Experimental study on the effect of abaca fibers on reinforced concrete: Evaluation of workability, mechanical, and durability-related properties. Fibers.

[149] Adesina A., Zhang J. (2024). Impact of concrete structures durability on its sustainability and climate resiliency. Next Sustain..

